# Broccoli Consumption Interacts with *GSTM1* to Perturb Oncogenic Signalling Pathways in the Prostate

**DOI:** 10.1371/journal.pone.0002568

**Published:** 2008-07-02

**Authors:** Maria Traka, Amy V. Gasper, Antonietta Melchini, James R. Bacon, Paul W. Needs, Victoria Frost, Andrew Chantry, Alexandra M. E. Jones, Catharine A. Ortori, David A. Barrett, Richard Y. Ball, Robert D. Mills, Richard F. Mithen

**Affiliations:** 1 Phytochemicals and Health Programme, Institute of Food Research, Norwich Research Park, Norwich, United Kingdom; 2 Department Farmaco-Biologico, School of Pharmacy, University of Messina, Messina, Italy; 3 School of Biological Sciences, University of East Anglia, Norwich, United Kingdom; 4 The Sainsbury Laboratory, Norwich Research Park, Norwich, United Kingdom; 5 Centre for Analytical Bioscience, School of Pharmacy, University of Nottingham, Nottingham, United Kingdom; 6 Department of Histopathology/Cytopathology, Norfolk and Norwich University Hospital, Norwich, United Kingdom; 7 Department of Urology, Norfolk and Norwich University Hospital, Norwich, United Kingdom; Fred Hutchinson Cancer Research Center, United States of America

## Abstract

**Background:**

Epidemiological studies suggest that people who consume more than one portion of cruciferous vegetables per week are at lower risk of both the incidence of prostate cancer and of developing aggressive prostate cancer but there is little understanding of the underlying mechanisms. In this study, we quantify and interpret changes in global gene expression patterns in the human prostate gland before, during and after a 12 month broccoli-rich diet.

**Methods and Findings:**

Volunteers were randomly assigned to either a broccoli-rich or a pea-rich diet. After six months there were no differences in gene expression between glutathione S-transferase mu 1 (*GSTM1*) positive and null individuals on the pea-rich diet but significant differences between *GSTM1* genotypes on the broccoli-rich diet, associated with transforming growth factor beta 1 (TGFβ1) and epidermal growth factor (EGF) signalling pathways. Comparison of biopsies obtained *pre* and *post* intervention revealed more changes in gene expression occurred in individuals on a broccoli-rich diet than in those on a pea-rich diet. While there were changes in androgen signalling, regardless of diet, men on the broccoli diet had additional changes to mRNA processing, and TGFβ1, EGF and insulin signalling. We also provide evidence that sulforaphane (the isothiocyanate derived from 4-methylsuphinylbutyl glucosinolate that accumulates in broccoli) chemically interacts with TGFβ1, EGF and insulin peptides to form thioureas, and enhances TGFβ1/Smad-mediated transcription.

**Conclusions:**

These findings suggest that consuming broccoli interacts with *GSTM1* genotype to result in complex changes to signalling pathways associated with inflammation and carcinogenesis in the prostate. We propose that these changes may be mediated through the chemical interaction of isothiocyanates with signalling peptides in the plasma. This study provides, for the first time, experimental evidence obtained in humans to support observational studies that diets rich in cruciferous vegetables may reduce the risk of prostate cancer and other chronic disease.

**Trial Registration:**

ClinicalTrials.gov NCT00535977

## Introduction

Prostate cancer is the most frequently diagnosed non-cutaneous cancer within the male population of western countries [1]. Epidemiological studies have suggested that diets rich in cruciferous vegetables, such as broccoli, may reduce the risk of prostate cancer [2], in addition to cancers at other sites [3]–[9] and myocardial infarction [10]. Some studies have specifically demonstrated that consuming one or more portions of broccoli per week can reduce the incidence of prostate cancer [11], and also the progression from localized to aggressive forms of prostate cancer [12]. The reduction in risk may be modulated by glutathione S-transferase mu 1 (*GSTM1*) genotype, with individuals who possess at least one *GSTM1* allele (i.e. approximately 50% of the population) gaining more benefit than those who have a homozygous deletion of *GSTM1* [11]. Our study investigates the mechanistic basis to the protective effect of broccoli and the interaction with *GSTM1* genotype.

**Figure 1:**
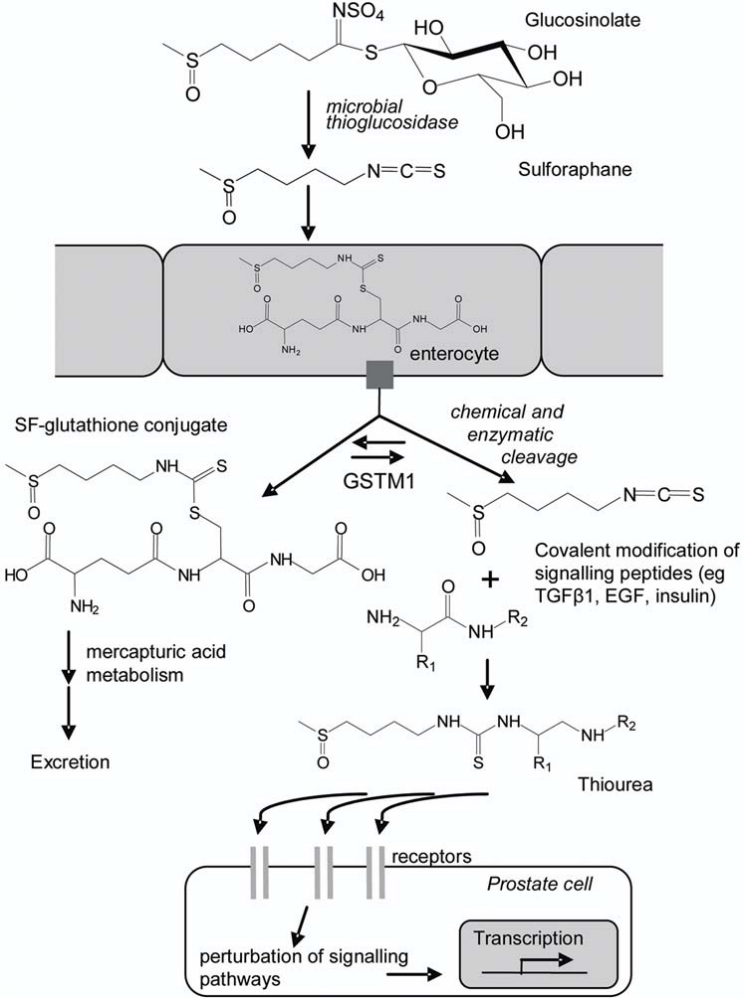
Metabolism of 4-methylsulphinylbutyl glucosinolate and sulforaphane. Upon entry into enterocytes sulforaphane (SF) is rapidly conjugated to glutathione, exported into the systemic circulation and metabolized through the mercapturic acid pathway. Within the low glutathione environment of the plasma the SF-glutathione conjugate may be cleaved, possibly mediated by *GSTM1*, leading to circulation of free SF in the plasma. This free SF can modify plasma proteins including signalling molecules, such as TGFβ, EGF and insulin.

Broccoli accumulates 4-methylsulphinylbutyl and 3-methylsulphinylpropyl glucosinolates in its florets, which are converted to the isothiocyanates (ITCs) sulforaphane (SF) and iberin (IB), respectively, either by plant thioglucosidases (‘myrosinases’) following tissue damage or, if the myrosinases have been denatured by cooking or blanching prior to freezing, by microbial thioglucosidases in the colon (Figure 1) [13]. These ITCs do not have the pungent flavour qualities associated with other dietary ITCs, such as those from mustards, rockets and watercress. SF and IB are passively absorbed by enterocytes, conjugated with glutathione and transported into the systemic circulation to be metabolized via the mercapturic acid pathway and excreted predominantly as N-acetylcysteine conjugates in the urine [14]. We previously demonstrated that following broccoli consumption, 45% of SF in the plasma occurs as free SF, as opposed to thiol conjugates, and that the peak concentration of SF and its thiol conjugates is less than 2 µM, falling to low (nM) levels within a few hours [14]. It was also shown that *GSTM1* null individuals excrete a higher proportion of SF via mercapturic acid metabolism than *GSTM1* positive individuals, and it was speculated that the remaining SF may be metabolized via an unknown pathway, and that this may account for the anticarcinogenic activity of broccoli.

Cell and animal studies have shown that SF is a potent inducer of phase 2 enzyme gene transcription, and, at higher concentrations, of cell cycle arrest and apoptosis, all consistent with anticarcinogenic activity [15]. However, these phenomena occur when cultured cells are exposed to considerably higher levels of SF (i.e. 10–100 µM for 24 h) than those found transiently in plasma after broccoli consumption (i.e. <2 µM for less than one hour). At these physiological concentrations, it is likely that any SF entering cells would be immediately conjugated with glutathione due to the relatively high intracellular glutathione concentration, with no effect on, for example, the Keap1-Nrf2 complex [16], [17]. Consistent with this hypothesis, there was no evidence for induction of phase 2 gene transcription in the gastric mucosa after an acute broccoli intervention [18].

In this study, we initially adopt an empirical approach in humans to elucidate the mechanisms that underlie the beneficial effects of a broccoli-rich diet, and explore the interaction with *GSTM1*. We compare and interpret global gene expression profiles in human prostate biopsy tissue before, during and after a 12 month broccoli-rich diet and a 12-month pea-rich diet. Subsequently, we provide a mechanistic explanation for how the observed changes in gene expression may be induced by normal dietary broccoli consumption.

## Methods

### Subjects and study design

Full details of the study protocol and the supporting CONSORT checklist and flow diagram are available as supporting information see Protocol S1, Checklist S1 and Figure S1. As stated in the protocol, it was intended to recruit forty men with a previous diagnosis of high-grade prostatic intraepithelial neoplasia (HGPIN), the pre-invasive *in situ* stage of prostatic adenocarcinoma, into a dietary intervention trial to study the effects of a diet rich in broccoli and a diet rich in peas on prostate gene expression. However, due to changes in clinical practice during the recruitment phase of the study there was marked reduction in the numbers of men diagnosed with HGPIN and it was not possible to recruit 40 men within the time constraints of the research programme. Thus, twenty-two male volunteers aged 57–70 years (Table 1) were recruited by a consultant urologist at Norfolk and Norwich University Hospital NHS Trust. Histological diagnosis was made by two consultant histopathologists, who had a special interest in prostate pathology. Ethical approval for the trial was obtained from the Norfolk Research Ethics Committee (reference 05/Q0101/9, see Text S1). All participants gave written, informed consent. Volunteers were excluded if they were undergoing chemopreventive therapy, were receiving testosterone replacement medication or 5 alpha reductase inhibitor, had active infection requiring treatment, had a body mass index (BMI) <18.5 or >35, or were diabetic. Subjects were allocated into a 12-month, parallel dietary intervention trial consisting of two dietary intervention groups: (i) consuming 400 g broccoli per week or (ii) consuming 400 g peas per week, in addition to their normal diet. The trial was conducted from April 2005–April 2007. Plasma prostate specific antigen (PSA) levels were quantified prior to the intervention study and after six and 12 months at the Norfolk and Norwich University Hospital with the use of a total PSA immunoassay. Volunteers avoided foods known to contain glucosinolates for 48 hours prior to each biopsy appointment to avoid acute effects.

**Table 1:** Volunteer characteristics and plasma PSA levels.

Age	BMI	GSTM1	PSA (ng/ml)		
			Pre-intervention	6-month	12-month
**Broccoli intervention**					
68	29	null	4.6	5.4	5.3
68	27	null	3.1	3.2	2.9
64	26	null	9.4	3.5	2.9
63	31	null	6.5	7.9	7.2
66	28	null	0.9	1.3	0.9
		**Mean (sd) 4.9 (3.25)**		**4.3 (2.50)**	**3.84 (2.44)**
57	27	positive	5.5	5.5	5.7
66	30	positive	13.6	16.8	13.4
69	27	positive	6.9	3.8	3.7
62	23	positive	2.2	2.2	1.9
59	29	positive	10.8	10.4	11.2
68	25	positive	9.7	12.5	7.2
64	32	positive	6.4	6.1	6.6
63	27	positive	7.9	9	10.8
		**Mean (sd) 7.9 (3.5)**		**8.3 (4.85)**	**7.56 (3.95)**
**Peas intervention**					
70	28	null	4.1	4.2	4.4
65	24	null	7.5	9.3	8.2
59	24	null	9.3	10.8	8
61	35	null	1.1	1.1	1.1
57	26	null	5.5	5.4	5.2
		**Mean (sd) 5.5 (3.15)**		**6.2 (3.92)**	**5.4 (2.92)**
70	23	positive	8.9	5.2	N/A*
61	29	positive	2.3	2.2	2.5
57	30	positive	3.5	5.5	4.9
		**Mean (sd) 4.9 (3.52)**		**4.3 (1.82)**	**3.7 (1.70)**

One of the 22 volunteers was diagnosed with prostatic adenocarcinoma at the study baseline biopsy (pre-intervention) and was removed from the study.

* This volunteer developed prostatic adenocarcinoma six months into the intervention and was removed from the study.

In addition to the transrectal ultrasound scan (TRUS)-guided needle biopsies of the prostate obtained from the volunteers immediately prior to the intervention study, and after six and twelve months, 18 benign and 14 malignant transurethral resection of the prostate (TURP) tissues were also obtained from the Norfolk & Norwich University Hospital *Partners in Cancer Research* Human Tissue Bank.

### Dietary intervention

Vegetables were delivered to the volunteers on a monthly basis. They were provided with a steamer and the volunteers were given a demonstration by the diet cooks at the Institute of Food Research of how to cook the vegetables. Portions of broccoli were steamed for 4–5 minutes and portions of peas were steamed for 2–3 minutes. Frozen peas (Birds Eye Garden Peas, http://www.birdseye.co.uk/) were purchased from a local retail outlet. To ensure consistency in glucosinolate content in frozen broccoli provided to the volunteers, the broccoli required for the intervention study was grown in one batch at an ADAS experimental farm at Terrington, near King's Lynn, UK (http://www.adas.co.uk/) and processed by Christian Salvesen (Bourne, Lincolnshire, UK, http://www.salvesen.co.uk/). It was blanched at 90.1°C for 74 s, frozen at −30°C and packaged into 100 g portions, then stored at −18°C until steamed by the volunteer. The broccoli was a high glucosinolate variety [19], [20]. The levels, mean (SD), of 4-methylsulphinylbutyl and 3-methylsulphinylpropyl glucosinolates (the precursors of SF and IB, respectively) were 10.6 (0.38) and 3.6 (0.14) µmolesg^−1^ dry weight, respectively, compared to 4.4 (0.12) and 0.6 (0.01) µmolesg^−1^ dry weight in broccoli purchased from local retail outlets. Although the level of glucosinolates were higher than standard broccoli, blanching prior to freezing denatured plant myrosinase, thus the levels of SF and IB derived from the high glucosinolate broccoli diet would be similar to or lower than those obtained from fresh broccoli with functional myrosinase. Levels of indole glucosinolates were similar in both high glucosinolate and standard broccoli.

### Compliance monitoring and dietary assessment

Volunteers completed weekly tick sheets during the 12-month intervention period to identify when the portions of vegetables were eaten. Every two weeks, volunteers were contacted by telephone and asked about adherence to the diet. A seven-day estimated food intake diet diary was completed by volunteers at baseline and after six months using household measures as an indication of portion size. Food intake from the diaries was inputted into Diet Cruncher v1.6.1 (www.waydownsouthsoftware.com/) and analyzed for differences in nutrient composition between the two intervention groups at baseline and six months after intervention.

### Genotyping

Genomic DNA was extracted from whole blood or from tissue samples using Qiagen QIAamp DNA minikit with RNase treatment according to the manufacturer's instructions (http://www.qiagen.co.uk/). *GSTM1* (NM_000561) genotype was determined using a real-time PCR procedure based on Covault and colleagues, using gene specific primers and probe and quantified relative to a two-copy gene control, a region in IVS10 of the breast cancer 1, early onset (*BRCA1*, NM_007294) gene [21]. Primers and probes were designed using Applied Biosystems Primer Express (http://www.appliedbiosystems.com/) and are given with PCR conditions in Table S1. Data were analyzed with Applied Biosystems Absolute Quantification software.

### RNA extraction and microarray hybridisation

Total RNA was isolated from the TURP tissue bank samples and the TRUS-guided needle biopsies from the volunteers with the use of QIAGEN® RNeasy mini kits according to the manufacturer's instructions (http://www.qiagen.co.uk/). The quantity of resulting RNA was measured using a spectrophotometer (Beckman). The RNA quality was determined using the Agilent 2100 Bioanalyzer (http://www.agilent.co.uk/). RNA samples from TURP biopsies of benign and malignant prostates and from TRUS-guided biopsies from both subject groups (peas and broccoli) at baseline, and at six and 12 months after intervention were hybridized onto Affymetrix Human U133 Plus 2.0 microarrays (http://www.affymetrix.com/) by the Nottingham Arabidopsis Stock Centre (NASC, http://arabidopsis.info/). Double-stranded cDNA synthesis and generation of biotin-labeled cRNA were performed according to the manufacturer's protocol (Affymetrix, http://www.affymetrix.com/). The final cRNA was checked for quality before fragmentation and hybridization onto the arrays.

One of the 22 volunteers was diagnosed with prostatic adenocarcinoma at the study baseline biopsy and was removed from the study. Eleven samples from the baseline biopsies, two samples from the six-month biopsies and three samples from the 12-month biopsies did not produce good quality RNA and/or sufficient cRNA and were not hybridized. In addition, one volunteer showed prostatic adenocarcinoma at the six-month biopsy; subsequent samples were removed from the study. Fluorescence intensity for each array was captured with a GeneChip® Scanner 3000 7G. Affymetrix GeneChip® Operating Software (GCOS) was used to quantitate each U133 Plus 2.0 array. Microarray data in this paper are compliant to the minimum information about a microarray experiment (MIAME) criteria and are deposited at Array Express (http://www.ebi.ac.uk/microarray-as/aer; Accession Number E-MEXP-1243).

### Microarray data analysis

Raw data files (CEL) were loaded into the DNA-Chip Analyzer software (dChip, http://biosun1.harvard.edu/complab/dchip/, build date September 2006) for normalization, generation of expression values and statistical analysis. Following normalization using the Invariant Set Normalization method, probe expression levels were calculated using the PM-only model. To identify genes that were changing between groups, different two-tailed *P*-value thresholds were applied calculated by Welch modified two-sample t-test in dChip. Paired or unpaired t-tests were performed as appropriate. To correct for multiple testing, False Discovery Rate (FDR) was estimated by permutation in dChip and the median of 100 permutations reported for each of the comparisons (1000 permutations on selected samples had little effect on FDR calculations). Unsupervised clustering was performed on benign and malignant samples using 1-Rank correlation as distance metric on a gene list of 3697 probes. These probes satisfied two criteria: first, that the coefficient of variation (CV) was between 0.5 and 1000; and secondly, that the percentage of Presence calls was more than 20% across all TURP benign and malignant samples.

For the purpose of sample classification, 19 laser-capture microdissected (LCD) epithelial cell microarrays (GEO Accession: GDS1439, http://www.ncbi.nlm.nih.gov/geo/) and 32 TURP benign and malignant microarrays were normalized together and model-based expression was calculated as described above in dChip. The LCD samples were derived from six benign prostate tissue samples, five clinically localized primary prostatic adenocarcinoma samples, two replicates of the five primary cancer samples after pooling, four metastatic prostatic adenocarcinoma samples and two replicates of the four metastatic prostate cancer samples after pooling [22]. Classification of the LCD epithelial cell samples was then performed using linear discriminant analysis (LDA) based on the TURP benign and malignant samples as training samples. LDA was performed using 442 probes that had higher than 100 units difference in signal intensity between TURP benign and malignant samples and were significantly different at *P*≤0.01 by Welch modified two-sample t-test.To identify pathways that are the most over-presented in the lists of differentially expressed genes, functional analyses using MAPPFinder and GenMAPP v2.1 were performed (http://www.genmapp.org/).

### Incubation of peptides with isothiocyanates

Incubations of SF or IB with bovine insulin (P01308, Sigma-Aldrich), recombinant human epidermal growth factor (EGF, P01133, R&D Systems, http://www.rndsystems.com/) and recombinant human transforming growth factor beta 1 (TGFβ1, P01137, R&D Systems) were performed in sodium phosphate-buffered saline solution (pH 7.4) or human blood plasma at 37°C for 0.5–24 h. Plasma was pre-treated by ultrafiltration to remove high molecular weight proteins (Microcon Ultracel YM-30 filter, MWCO 30,000). Samples were either analyzed directly by LC-MS/MS or by LC-MS/MS analysis of tryptic digests of gel electrophoresis bands.

### Direct LC-MS/MS analysis of peptides incubated with isothiocyanates

The LC system used was a Shimadzu series 10AD VP (Shimadzu, http://www.shimadzu.com/). The column was an ACE 300 C18, 150×2.1 mm (5 µm particle size) used at 40°C. Mobile phase A was 0.1% formic acid in water, mobile phase B, 0.1% formic acid in acetonitrile and the flow rate was 0.25 ml/min. A linear gradient was used from 25% B to 35% B over 0 to 5 min, then a further gradient from 35% B to 99% B over 6 min followed by 99% B for 4 min. The column was re-equilibrated for a total of 3 min. The injection volume was between 5–20 µl. All MS experiments were conducted on a 4000 QTRAP hybrid triple-quadrupole linear ion trap mass spectrometer using Analyst version 1.4.1 software (Applied Biosystems, http://www.appliedbiosystems.com/) equipped with a TurboIon source used in positive ion electrospray mode. The probe capillary voltage was optimized at 4200 V, desolvation temperature set to 400°C, curtain gas, nebulizing and turbo spray gas were set to 40, 10 and 20, respectively (arbitrary values). Declustering potential was ramped between 50–120 V. Nitrogen was used for collisionally induced dissociation (CID). The peak-width was set on Q1 and Q3 at 1.0 Th (measured at half height) for all MS and MS/MS experiments. Spectra were obtained over the range *m/z* 800–2000 with scan times of 1–2 sec. Operating in LIT mode Q0 trapping was activated and dynamic fill time used, the scan rate was set to 250 Th/s for enhanced product ion (EPI) scans, excitation time was 150 msec, excitation energy 25 V and entry barrier 4 V. For EPI spectrum acquisition the precursor ions of interest for conjugates of SF with insulin (*m/z* 1183.9 MH_5_^5+^), EGF (*m/z* 1088.8, MH_5_^6+^) and TGFβ (*m/z* 1981.9, MH_5_^13+^) were selected, the collision energy was ramped between 30–120 V and spectra were obtained over the range *m/z* 100–1500 with a scan time of 1.9 sec. MS^3^ settings were identical to MS^2^ except that the collision energy was 50–80 V and declustering potential was 50–80 V.

### LC-MS/MS analysis of TGFβ1 incubated with SF following electrophoresis and tryptic digestion

1 µg aliquots of the TGFβ1 protein, supplied with bovine serum albumin as carrier, were incubated with either DMSO or 1.2 µmoles of SF for 30 minutes at 37°C and run onto denaturing 4–12% Bis-Tris NuPAGE gels (Invitrogen, http://www.invitrogen.com). Bands were excised and digested with trypsin (Promega, http://www.promega.com/) after reduction with dithiothreitol (DTT) and alklyation with iodoacetamide (Sigma-Aldrich, http://www.sigmaaldrich.com/). Extracted peptides were lyophilized and re-dissolved in 1% acetonitrile, 0.1% formic acid for analysis by mass spectrometry. LC-MS/MS analysis was performed using a LTQ mass spectrometer (Thermo Electron Corporation, http://www.thermo.com/) and a nanoflow-HPLC system (Surveyor, Thermo Electron). Peptides were applied to a precolumn (C18 pepmap100, LC Packings, http://www.lcpackings.com/) connected to a self-packed C18 8-cm analytical column (BioBasic resin ThermoElectron; Picotip 75 µm id, 15 µm tip, New Objective, http://www.newobjective.com/). Peptides were eluted by a gradient of 2 to 30% acetonitrile in 0.1% formic acid over 40 min at a flow rate of approximately 250 nL min^−1^. Data-dependent acquisition of MS/MS consisted of selection of the five most abundant ions in each cycle: MS mass-to-charge ratio (m/z) 300 to 2000, minimum signal 1000, collision energy 25, 5 repeat hits, 300 sec exclusion. In all cases the mass spectrometer was operated in positive ion mode with a nano-spray source and a capillary temperature of 200°C, no sheath gas was employed; the source voltage and focusing voltages were optimized for the transmission of angiotensin. Raw data were processed using BioWorks 3.3 (Thermo Electron Corporation). Searches were performed with Mascot (Matrix Science, http://www.matrixscience.com/) against SPtrEMBL (4719335 sequences) restricted by taxonomy to *Homo sapiens* (68982 sequences), oxidized methionine and carbamidomethyl cysteine residues were allowed as variable modifications as was putative SF. The error tolerance of the parent ion was ±1.2 Da and the fragment mass tolerance was ±0.6 Da, one missed cleavage was permitted. Error tolerant searches in Mascot against TGFβ were routinely performed and extracted ion chromatograms and manual inspection of spectra were prepared using Qual Browser and BioWorks 3.3 (Thermo Electron Corporation).

### Luciferase reporter gene assay

NIH 3T3 cells stably transfected with a CAGA12-luc plasmid, which responds to Smad activation [23], were cultured in DMEM supplemented with 10% fetal calf serum (FCS), 1% penicillin, 1% streptomycin, 1% L-glutamine and 0.4 mg/ml geneticin. Cells were seeded into complete growth medium in a six-well tissue culture dish for 24 h, after which the medium was replaced with low serum medium (0.5% FCS) containing one of three treatments: (1) TGFβ1 (to achieve a final concentration of 2 ng ml^−1^) in PBS buffer, (2) TGFβ1+10 mM DTT in PBS buffer (3) TGFβ1+2 µM SF in PBS buffer. To simulate SF pharmacokinetics, all test samples were incubated at 37°C for 30 minutes prior to dialysis, performed in PBS buffer for 4 hours using Slide-A-Lyzer Dialysis Cassettes MWCO 3.5K (PIERCE, http://www.piercenet.com/). Dialysis reduced SF concentration to 34 nM. As additional controls, cells were treated with PBS without TGFβ1 and PBS with SF (34 nM). The luciferase activity was determined 16 h following treatment using the Luciferase Reporter Gene assay (Roche Applied Science, http://www.roche.com/) in a Perkin Elmer Wallac Victor 2 1420 multilabel counter plate reader (http://las.perkinelmer.com/). Briefly, cells were washed twice with PBS and lysed in cell lysis buffer supplied with the assay. Chemiluminescence was immediately quantified following the addition of luciferase assay substrate. Luciferase values were normalized to protein concentration quantified using the BCA assay (Sigma-Aldrich, http://www.sigmaaldrich.com/). The experiment was repeated four times, with three replicates of each treatment per experiment. Statistical analysis was performed using 1-way ANOVA with the statistical software, R [24].

## Results

### Comparison of gene expression of benign and malignant TURP tissue samples

**Figure 2:**
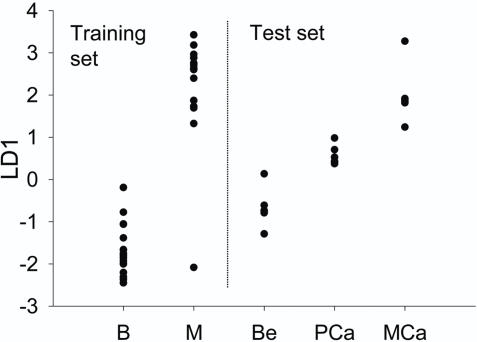
LDA of an independent prostate microarray data set. Linear discriminant analysis (LDA) using the benign (B) and malignant (M) TURP prostate tissue for this study as training samples to classify the laser-capture microdissected (LCD) epithelial prostate cell samples (GEO Accession:GDS1439), consisting of benign (Be), primary cancer (PCa) and metastatic cancer (MCa) samples. LDA was performed on a gene list that distinguished the benign and malignant TURP samples as described in Methods. Here, the first linear discriminant (LD1) is shown.

We compared global gene expression profiles in surgically resected benign and malignant prostate TURP tissue using RNA extracted from heterogeneous tissue (such as we intended to use in the intervention study). Unsupervised clustering distinguished unambiguously the benign and malignant samples (data not shown). Pathway analyses for genes that were significantly different between the two groups were undertaken with the use of GenMapp software, and identified pathways that are frequently reported to be perturbed during carcinogenesis (Tables 2a and 3a). To validate further our methods of data analysis and to determine whether microarray data from gross heterogeneous tissue are comparable to data generated from LCD epithelial cells, we analyzed independent data sets of LCD epithelial cells (GEO Accession: GDS1439) from benign, localized and metastatic prostate cancer. We used our benign and malignant samples as a training set for linear discriminant analyses (LDA) and the independent data as test sets, and found that the LDA model correctly distinguished the benign, localized and metastatic LCD epithelial cell samples (Figure 2). Thus, this preliminary study provides validation for our approach to the statistical analyses of array data.

**Table 2:** Pathway analyses of prostate biopsy tissue.

Pathway	Genes changed/ Genes on MAPP	Adjusted *P*-value*
**a. Benign compared with malignant TURP tissue**		
Focal adhesion	57/187	<0.001
TGFβ receptor	47/151	0.002
Circadian exercise	20/48	0.006
Fatty acid metabolism	24/80	0.012
Prostaglandin synthesis regulation	14/31	0.026
Actin binding	53/213	0.028
GPCRs Class A rhodopsin-like	15/262	0.05
**b. ** ***GSTM1*** ** positives compared with ** ***GSTM1*** ** nulls ** ***post*** ** six month broccoli intervention**		
EGFR1	76/177	<0.001
Adipogenesis	52/130	0.026
TGF-beta receptor	58/151	0.039
**c. Paired samples pre and ** ***post*** ** 12 month dietary intervention**		
**0–12 month peas**		
Androgen receptor	18/112	0.042
**0–12 month broccoli ** **		
mRNA processing	40/125	<0.001
Androgen receptor	33/112	<0.001
TGFβ receptor	39/151	0.004
Insulin signalling	38/159	0.014
Delta-notch	24/ 85	0.019
Wnt signalling	28/109	0.02
EGFR1	40/177	0.02
IL-2	21/ 76	0.036

EGFR, epidermal growth factor receptor; GPCRs, G-protein coupled receptors; IL-2, interleukin 2; TGFβ, transforming growth factor beta; TURP, transurethral resection of the prostate; Wnt, wingless-type MMTV integration site. Pathways in GenMAPP that are enriched in the gene lists that differentiate groups are shown. Only pathways with adjusted *P* values ≤0.05 are shown. Also, the number of genes changing between groups that belong to these pathways is shown alongside the total number of genes that constitute the pathway. Pathway analysis was performed on gene lists generated in dChip that were statistically significant (*P*≤0.05, Welch modified two-sample paired or unpaired t-test) between the two groups. No fold cutoff was used. For details on gene lists see Table 3.

* *P*-values were calculated in GenMAPP using a non-parametric statistic based on 2000 permutations of the data and further adjusted for multiple testing by Westfall-Young adjustment.

** *GSTM1* positive volunteers, n = 4.

**Table 3:** Differentially expressed probes in prostate tissue.

	Fold change	*P*<0.05*	*P*<0.005*	*P*<0.0005*
**(a) Differences between benign and malignant TURP tissue**				
Benign v Malignant	>1.0	3810 (353)	683 (7)	124 (0)
	>1.5	1081 (59)	400 (2)	104 (0)
	>2.0	277 (7)	140 (0)	54 (0)
**(b) Differences between GSTM1 positive and null genotypes**				
Benign (TURP)	>1.0	661 (538)	19 (17)	0 (0)
	>1.5	160 (186)	7 (14)	0 (0)
	>2.0	50 (40)	1 (3)	0 (0)
Malignant (TURP)	>1.0	686 (431)	8 (13)	0 (0)
	>1.5	244 (152)	4 (8)	0 (0)
	>2.0	44 (33)	1 (3)	0 (0)
Pre-intervention	>1.0	730 (484)	26 (9)	0 (0)
	>1.5	252 (79)	16 (3)	0 (0)
	>2.0	43 (9)	8 (1)	0 (0)
6 month broccoli	>1.0	7976 (351)	434 (4)	17 (0)
	>1.5	2790 (91)	268 (2)	14 (0)
	>2.0	316 (8)	31 (0)	1 (0)
6 month peas	>1.0	220 (220)	6 (4)	0 (0)
	>1.5	33 (41)	5 (3)	0 (0)
	>2.0	5 (10)	1 (1)	0 (0)
**(c) Differences between paired samples**				
0–12 months broccoli	>1.0	2857 (96)	151 (0)	1 (0)
	>1.5	1243 (12)	141 (0)	1 (0)
	>2.0	213 (0)	62 (0)	1 (0)
0–12 months peas	>1.0	1199 (42)	19 (0)	0 (0)
	>1.5	495 (18)	19 (0)	0 (0)
	>2.0	81 (1)	4 (0)	0 (0)

Probe numbers that have satisfied the comparison criteria of fold change and *P*-value cutoffs are shown. Numbers in parentheses represent the median false discovery rate calculated in dChip after 100 permutations of the samples.

* *P*-values were calculated in dChip by a Welch modified two-sample t-test. n = 18 for Benign, n = 14 for Malignant; n = 4 for GSTM1(+) Benign, n = 14 for GSTM1(−) Benign; n = 5 for GSTM1(+) Malignant, n = 9 for GSTM1(−) Malignant; n = 7 for GSTM1(+) Pre-intervention, n = 3 for GSTM1(−) Pre-intervention; n = 6 for GSTM1(+) 6-month broccoli, n = 5 for GSTM1(−) 6-month broccoli; n = 3 for GSTM1(+) 6-month pea and n = 5 for GSTM1(−) 6-month pea intervention.

** *P*-values were calculated in dChip by a Welch modified two-sample paired t-test, n = 4 for each of the diet interventions.

### Variation in plasma PSA levels

PSA levels prior to the intervention were in similar range to that previously reported for men of an equivalent age range diagnosed with HGPIN [25]. There was no significant association with GSTM1 genotype, and no consistent changes in PSA levels after six or 12 months within either arm of the intervention study (Table 1).

### Differences in global gene expression between *GSTM1* positive and null individuals

**Figure 3:**
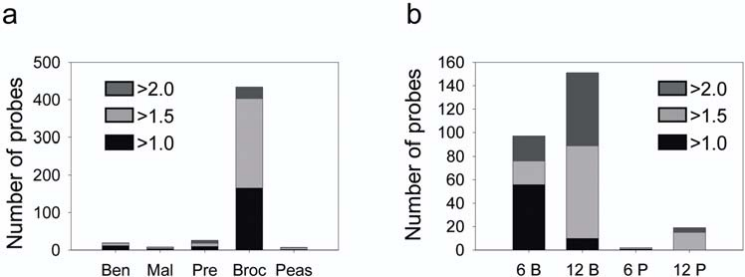
Effect of dietary intervention on gene transcription. a, Number of probes that differ between *GSTM1* positive and null genotypes (*P*≤0.005, Welch modified two-sample t-test) in TURP tissue from benign (Ben) and malignant (Mal) prostates, and TRUS-guided biopsy tissue from volunteers at pre-intervention (Pre), post 6 months broccoli-rich diet (Broc) and post 6 months pea-rich diet (Peas). b, Number of probes that differ between pre-intervention TRUS-guided biopsy samples and after 6 months broccoli (6B)-, 6 month pea (6P)-, 12 month broccoli (12B)- and 12 month pea (12 P)-rich diets (*P*≤0.005, Welch modified two-sample paired t-test). Shading correspond to different fold cutoffs applied. See Table 2 for full details of probe numbers, *P*-values and median false discovery rates.

We initially genotyped the resected TURP tissue samples and compared gene expression profiles between *GSTM1* positive and null genotypes within the benign and malignant samples. We found few differences between genotypes, with similar high median false discovery rates (Figure 3a, Table 3b). Likewise, we compared gene expression profiles obtained from needle biopsies of the prostate from *GSTM1* positive and null men who had previously been diagnosed with HGPIN and found few differences.

We then compared gene expression profiles between *GSTM1* genotypes in needle biopsy tissue of twenty-one men who had been recruited into the dietary intervention study. Eight of the men within this study had been asked to consume 400 g of steamed frozen peas per week, and the other thirteen were requested to consume 400 g of steamed frozen broccoli per week, but otherwise to consume their normal diet. Diet was assessed with seven-day diet diaries prior to the intervention and after six months. No significant differences were found in diet components, apart from the consumption of broccoli and peas (Table 4). We found many differences in the prostate gene expression between *GSTM1* positive and null men who had been on the broccoli diet for six months, but few, if any, differences in gene expression between *GSTM1* positive and null men who had been on the pea diet (Figure 3a, Table 3b). To investigate the potential consequences of the differences in gene expression between *GSTM1* genotypes following the broccoli-rich diet, we analyzed these data via GenMapp. Three pathways, EGF receptor, adipogenesis and TGFβ receptor, were identified in which genes occurred at a higher frequency than they would by chance (Table 2b). The relative expression of genes between the *GSTM1* positive and null men in the EGF receptor and TGFβ receptor pathways are given as supporting information (Table S2 and S3).

**Table 4:** Dietary analysis of average daily intakes of nutrients.

Variable	Baseline	6 months	*P*-value*
**Pea-rich diet (n = 7)**			
Fat (g)	93.76 (32.97)	90.99 (25.83)	0.831
Protein (g)	87.50 (15.15)	95.77 (33.31)	0.526
CHO (g)	240.04 (79.85)	242.94 (72.97)	0.872
Energy (KJ)	9081.88 (2480.80)	9506.00 (2405.38)	0.738
Alcohol (g)	12.65 (9.87)	25.07 (35.42)	0.415
Cholesterol (mg)	353.57 (74.43)	345.29 (201.06)	0.930
Vitamin C (mg)	81.00 (66.05)	79.43 (62.58)	0.846
Vitamin E (mg)	8.24 (5.64)	7.15 (3.60)	0.395
Vitamin D (µg)	3.77 (3.25)	3.84 (1.76)	0.949
β-Carotene (mg)	1.94 (1.57)	3.22 (2.29)	0.184
Folate (µg)	261.00 (89.36)	332.86 (214.50)	0.489
Iron (mg)	11.73 (4.36)	13.32 (4.92)	0.555
Selenium (µg)	50.14 (13.01)	49.14 (23.08)	0.923
Peas (g)	8.57 (10.23)	57.41 (18.86)	**0.001**
Broccoli (g)	18.49 (30.89)	9.90 (13.61)	0.431
Estimated GSL (µmol)	9.36 (15.63)	5.01 (6.88)	0.431
**Broccoli-rich diet (n = 11)**			
Fat (g)	90.93 (29.27)	91.57 (33.70)	0.929
Protein (g)	96.99 (20.02)	96.97 (21.26)	0.996
CHO (g)	276.28 (76.03)	296.18 (72.99)	0.305
Energy (KJ)	9633.45 (2311.35)	9980.73 (2286.62)	0.488
Alcohol (g)	9.75 (7.20)	10.48 (9.70)	0.841
Cholesterol (mg)	337.27 (168.29)	298.46 (123.99)	0.211
Vitamin C (mg)	262.55 (175.83)	303.00 (188.52)	0.590
Vitamin E (mg)	11.31 (5.73)	11.14 (4.82)	0.924
Vitamin D (µg)	5.22 (3.17)	3.65 (1.08)	0.076
Beta Carotene (mg)	4.07 (3.01)	3.63 (2.53)	0.667
Folate (µg)	477.82 (188.20)	491.36 (193.47)	0.762
Iron (mg)	14.29 (2.09)	14.09 (2.25)	0.916
Selenium (µg)	77.09 (29.89)	68.82 (26.22)	0.304
Peas (g)	4.16 (5.51)	7.60 (8.46)	0.227
Broccoli (g)	25.89 (24.49)	55.84 (7.71)	**0.002**
Estimated GSL µmol	13.10 (12.39)	79.30 (10.94)	**<0.0001**

Variables shown are given in mean (sd) units per day. GSL refers to the glucosinolate precursors of sulforaphane and iberin (ie 4-methylsulphinylbutyl and 3-methylsulphinylpropyl glucosinolate) respectively. Similar analysis between GSTM1 positive and null individuals showed no difference in dietary intakes after 6 months within either broccoli-rich or pea-rich intervention.

* *P*-values were calculated in Minitab using a paired t-test.

### Changes in gene expression before and after the dietary intervention

We used paired t-tests to identify genes that had changed in expression between 0 and 6 months and 0 and 12 months in biopsy samples from individuals within each arm of the intervention to quantify changes in expression with time. Within the broccoli arm, we restricted analyses to *GSTM1* positive individuals. We found after both 6 months and 12 months there were more changes in expression within the broccoli-rich arm than the pea-rich arm (Figure 3b, Table 3c). Pathway analyses with genes that changed in expression between 0 and 12 months identified changes only in the androgen receptor pathway in the pea-rich arm, while in the broccoli-rich arm androgen receptor pathway was identified, along with several other signalling pathways, including insulin signalling, TGFβ and EGF receptor pathways (Table 2c, Table S4 and S5). Analyses with genes that changed in expression between 0 and 6 months in the broccoli arm also identified changes in TGFβ receptor pathway (adjusted *P* = 0.001), insulin signalling (adjusted *P* = 0.035) and EGF receptor signalling (adjusted *P* = 0.068).

Thus, evidence for the effect of broccoli consumption on modulation of TGFβ and EGF signalling has been obtained in two independent analyses: Firstly, the comparison of gene expression profiles of GSTM1 positive and null individuals who had consumed the broccoli-rich diet for six months, and, secondly, the paired analyses of gene expression profiles from biopsies obtained at 0 and 12 months from GSTM1 positive individuals who had consumed the broccoli-rich diet. It is important to note that these analyses do not share any array data sets.

### Chemical interactions between TGFβ1, insulin and EGF peptides and broccoli isothiocyanates

**Figure 4:**
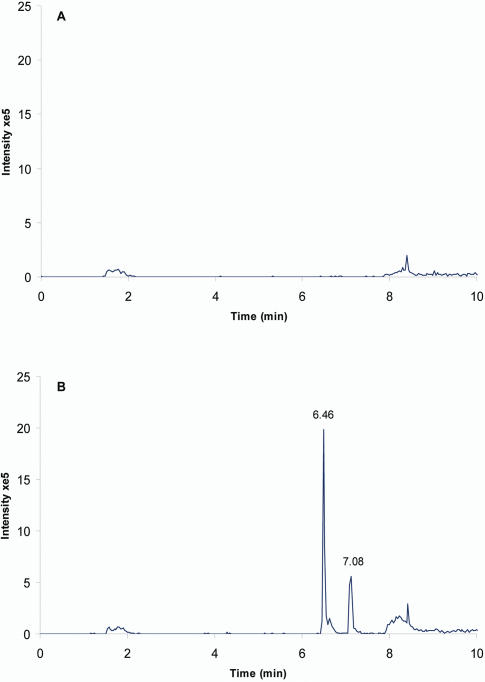
LC-MS of insulin incubated with and without SF in human plasma. Extracted ion LC-MS chromatograms (*m/z* 1183.6–1184.1) of insulin-SF MH_5_^5+^ in (A) unmodified insulin (20 µg/ml) in human plasma control and (B) human plasma incubated with insulin (20 µg/ml) and 50 µM SF for 4 h at 37°C, showing the appearance of two different insulin-SF conjugates at retention times of 6.46 and 7.08 min. The enhanced product ion (EPI)-MS spectra of these two insulin-SF conjugates are shown in Figure 5.

**Figure 5:**
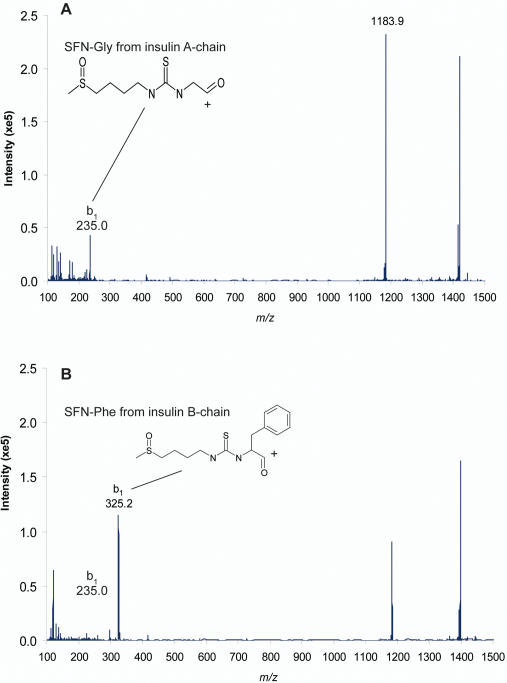
Enhanced product ion (EPI)-MS spectra of the two insulin-SF conjugates. MS^2^ product ion spectra of (A) 6.46 min and (B) 7.08 min retention time peaks from LC-MS analysis of human plasma incubated with bovine insulin and 50 µM SF for 4 h at 37°C. In (A) and (B) *m/z* 1183.9 corresponds to insulin-SF MH_5_^5+^ and in (A) *m/z* 235.0 corresponds to Gly-SF, the N-terminal amino acid of insulin A chain and in (B) *m/z* 325.2 corresponds to Phe-SF, the N-terminal amino acid of insulin B chain.

Having demonstrated that broccoli consumption modulates several cell signalling pathways, we sought a mechanistic explanation. Incubation of insulin, EGF and TGFβ1 peptides with the isothiocyanates SF or IB in PBS pH 7.4 at 37°C for a period of 0.5 to 24 hours gave consistent evidence of the formation of a covalently bound conjugate of the respective peptide and the ITC. This was further investigated for physiological relevance by performing the same incubations in human plasma depleted of high MW proteins. LC-MS/MS analysis showed the appearance of one or more additional LC-MS peaks when SF or IB were incubated with the peptides. For example, in Figure 4 an extracted ion chromatogram (*m/z* 1183.9, corresponding to insulin- SF MH_5_^5+^) shows the appearance of two insulin-SF conjugates compared with the control incubation. MS^2^ analysis of these peaks (Figure 5) confirmed the presence of two diagnostic fragment ions at *m/z* 235 and *m/z* 325 corresponding to the addition of SF to the two N-terminal amino acids of insulin Gly-SF and Phe-SF. Similar results were obtained to identify Gly-IB (*m/z* 221) and Phe-IB (*m/z* 311) from the incubation (data not shown). Comparable evidence was obtained for the formation of EGF conjugates with SF in human plasma corresponding to the addition of SF to the N-terminal asparagines residue (*m/z* 309) of EGF.

**Figure 6:**
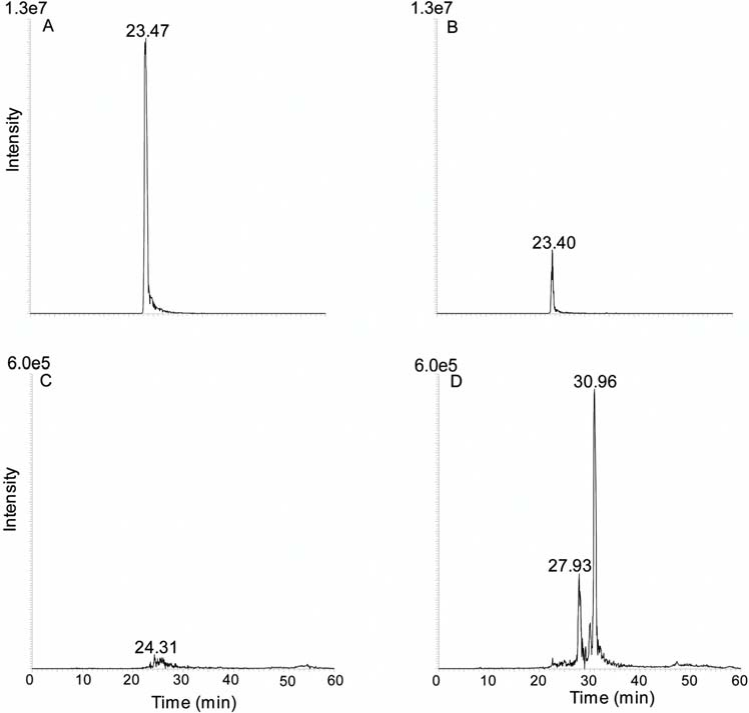
LC-MS of TGFβ1 incubated with and without SF. Extracted ion chromatograms (MS) of precursor masses representing the unmodified N-terminal peptide of TGFβ1 (*m/z* 768.5) and the modified N-terminal peptide (*m/z* 877.2) A of *m/z* 768.2–769.2 from DMSO treated TGFβ1, B of *m/z* 768.2–769.2 from SF treated TGFβ1, C of *m/z* 876.7–877.7 DMSO treated TGFβ1 and D of *m/z* 876.7–877.7 SF treated TGFβ1.

**Figure 7:**
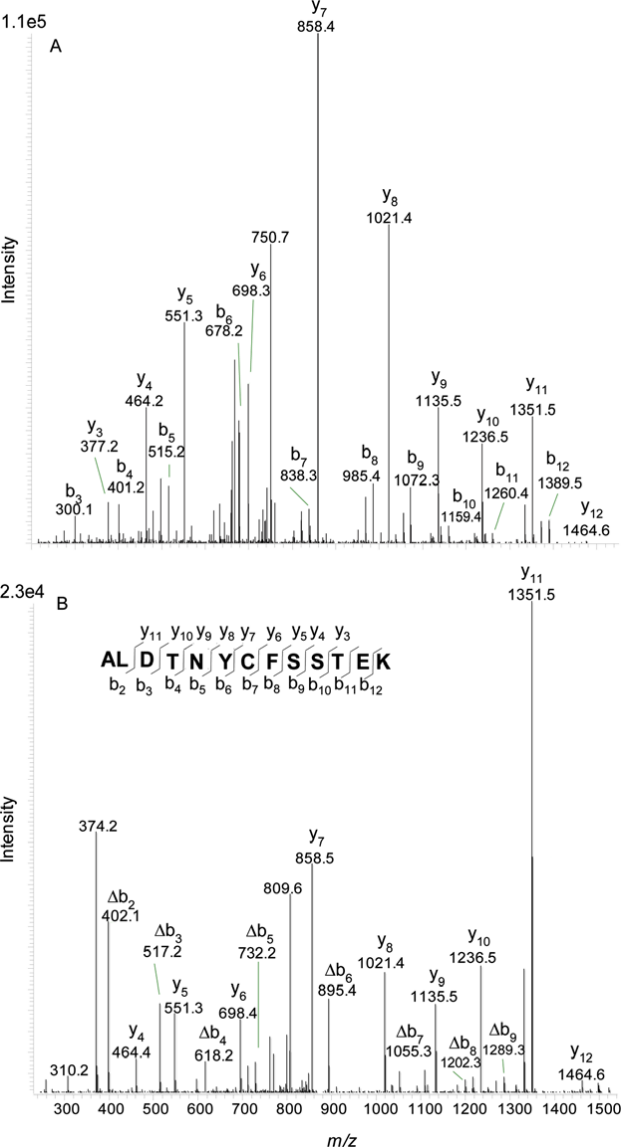
N-terminal modification of TGFβ1 by SF. MS/MS spectra of *m/z* 768.7 representing the unmodified N-terminal peptide of TGFβ1 at retention time 23.43 min (A) and *m/z* 877.2 representing a modified form of TGFβ1 seen only in SF treated samples at retention time 30.85 minutes (B). Note that the y ion series remains the same while the b ion series shifts (Δ) indicating an N-terminal modification of mass 217±0.8 Da. Figure S2 provides an explanation of the mass addition of 217, as opposed to 177.

To provide additional information of modifications to TGFβ1, we adopted a complementary approach. 1 µg aliquots of the protein were incubated with either DMSO or 1.2 µmoles of SF for 30 minutes at 37°C and separated by SDS-PAGE electrophoresis. Bands were excised and digested with trypsin before analysis by LC-MS/MS. TGFβ1 was robustly identified in bands of 25 kDa corresponding to the active dimer. The N-terminal peptide ALDTNYCFSSTEK was identified from parent ion *m/z* 768.5 in both DMSO (control) and SF-treated samples (Figure 6). A precursor ion *m/z* 877.2 was observed only in SF treated samples. MS/MS analysis of both precursor ions revealed a strong series of fragment peaks that were common to both (Figure 7) precursor ions. These fragmentation patterns are consistent with the unmodified y ion series for the peptide ALDTNYCFSSTEK (including carbamidomethyl cysteine +57) and a b ion series shifted by 217.4±0.8 Da in the SF-treated sample. These results strongly support an N-terminal modification to TGFβ1 by SF. Addition of SF would result in a mass addition of 177, as observed with LC-MS analyses of intact TGFβ1, as described above. It is highly likely that the addition of 217, as opposed to 177, is due to subsequent reaction of the thiourea with iodoacetamide, added to the reaction mixture to alkylate reduced disulphide linkages, to result in a mixture of isomeric carbamimidoylsulfanylacetamides, which undergo cyclisation and loss of NH_3_ to give the corresponding iminothiazolidinones (Figure S2).

### Enhancement of TGFβ1 signalling after pre-incubation with sulforaphane

**Figure 8:**
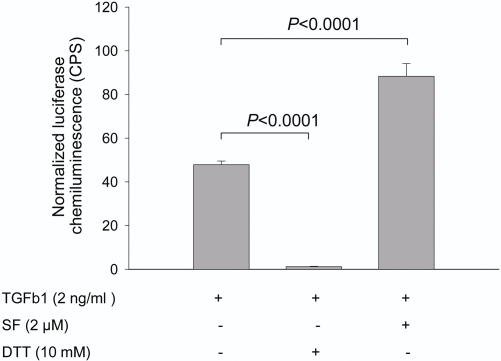
Activation of TGFβ1/Smad mediated transcription by SF. NIH3T3 cells containing a CAGA12-luc plasmid were treated with TGFβ1 alone, TGFβ1 and 10 mM DTT, which disrupts the active TGFβ1 dimer, or TGFβ1 and 2 µM SF. All samples were pre-incubated for 30 minutes and further dialyzed for 4 h so that the final concentration of SF was 34 nM. As an additional negative control cells received no treatment or only 34 nM SF, both of which failed to induce luciferase. Chemiluminescence was normalized to the protein concentration of each sample (for details see Methods). This is a representative experiment of a total of four similar experiments performed. Data shown are mean (s.e.m) of three replicates.

As thiourea derivatives of proteins by isothiocyanates have been shown to modify physicochemical and enzymatic properties [26], [27], we sought to assess whether SF modification of extracellular signalling proteins had functional consequences. We focussed on TGFβ1 signalling due to its profound role in maintaining tissue homoeostasis through controlling cell proliferation and behaviour [28], [29]. TGFβ1-induced Smad-mediated transcription was quantified in NIH3T3 cells stably transfected with a CAGA12-luc plasmid, in which luciferase activity can be measured upon activation of Smad proteins [30]. Exposure of cells to TGFβ1 induced luciferase activity as expected. When cells were exposed to TGFβ1 that had been pre-incubated with physiologically appropriate concentrations of SF (2 µM) for 30 minutes followed by dialysis, to simulate SF plasma pharmacokinetics [14], there was an increase in Smad-mediated transcription compared to exposure to TGFβ1 alone (Figure 8). Exposure of cells to the residual SF (34 nM) did not result in enhanced transcription suggesting that SF induces Smad activation indirectly, consistent with our previous observation that SF binds to the ligand itself. It is also conceivable that SF may interact with the extracellular domain of the receptor to alter downstream signalling.

## Discussion

To our knowledge, this is the first dietary intervention study to analyse global gene expression profiles within a target tissue before and after a 12 month intervention, and to stratify gene expression profiles by genotype. While we do not observe any consistent changes in plasma PSA levels over the 12 month period of the intervention, we were able to quantify extensive changes in gene expression. We find little evidence to support potential mechanisms derived from animal and cell models to explain the observational data that consuming broccoli may reduce risk of cancer, but considerable evidence for the perturbation of several signalling pathways that are associated with carcinogenesis and inflammation (Table 2b and c). It is possible that the net effect of perturbation of these pathways may reduce the risk of cell proliferation, and maintain cell and tissue homoeostasis. However, whilst quantification of gene expression and pathway analyses provides information concerning which pathways may be modified by time or diet, it can provide little information about the precise nature of how these pathways are perturbed. This requires further analysis of mRNA and protein turnover, and *post* translational protein modifications such as phosphorylation, associated with components of the signal transduction pathway and downstream targets. Furthermore, the effect of pathway perturbation may depend upon the precise cell type, with potentially differential effects occurring in epithelial and stromal cells. Despite these reservations, it is of considerable interest that broccoli intervention is associated with perturbation of TGFβ1, EGF and insulin signalling, each of which has been associated with prostate carcinogenesis [31]–[35], in addition to carcinogenesis at other sites [28], [36], [37], and inflammation associated with myocardial infarction [38]. It is noteworthy that broccoli consumption was also associated with alterations in mRNA processing, and this is being further explored.

It is likely that the major bioactive products derived from broccoli are the isothiocyanates, sulforaphane and iberin. These have been shown to have a multitude of biological activities in cell models consistent with anticarcinogenic activity [15]. However, these studies largely involve exposing cells to concentrations of SF and IB far in excess of those which occur transiently in the plasma after broccoli consumption, and are mediated by the intracellular activity of the ITCs by, for example, perturbing intracellular redox status, depletion of glutathione and perturbation of the Keap1-Nfr2 complex. We question whether these processes would occur *in vivo*, as any of the ITCs entering cells would immediately be inactivated through conjugation with glutathione that would be present in relatively high concentration. Thus, we explored whether the biological activity of ITCs may be mediated through their chemical interaction with signalling peptides within the extracellular environment of the plasma, which has a low glutathione concentration. We demonstrated that ITCs readily form thioureas with signalling proteins in the plasma through covalently bonding with the N-terminal residue. It is likely that ITCs chemically react with other plasma proteins and a global analysis of plasma protein modifications by ITCs is warranted. It is also possible that other types of chemical modification of plasma proteins by ITCs may occur, such as covalent bonding through cysteine and lysine residues [39], [40].

Previous studies have shown that isothiocyanate-derived thioureas modify the physicochemical and enzymatic properties of the parental proteins [26], [27]. Thus, it is possible that the perturbation of signalling pathways in the prostate is mediated by protein modifications that occur in the extracellular environment. We provide further evidence for this hypothesis by demonstrating that pre incubation of TGFβ1 with a physiological appropriate concentration of SF (2 µM for 30 minutes), followed by dialysis for 4 h to simulate SF pharmacokinetics, results in enhanced Smad-mediated transcription. As TGFβ1/Smad-mediated transcription inhibits cell proliferation in non-transformed cells [31], [41], the enhancement of Smad-mediated transformation by SF would be consistent with the anticarcinogenic activity of broccoli, in addition to reduced risk of myocardial infarction [10], [38]. In certain circumstances, enhancement of TGFβ signalling has been associated with tumour progression within already initiated cells, although the precise pathways by which this is mediated have not been fully resolved [42]. To what extent a broccoli-rich diet may influence these processes requires further studies. However, we consider it likely that it is the net effect of changes in several pathways, as opposed to just TGFβ1, which may underlie the observed reduction in both cancer and myocardial infarction through broccoli/crucifer consumption.

A previous study has demonstrated that isothiocyanates can inhibit EGF signalling, but without a mechanistic explanation [43]. In the current study, we show that SF will bind to the EGF ligand, and this may underlie our results and those reported previously [43]. Moreover, chemical modification of signalling proteins by ITCs may be complemented by modification of receptor proteins, as has previously been shown for the TRPA1 receptor [39], [40].

Perturbation of signalling pathways is additionally determined by *GSTM1* genotype. The interaction between diet and *GSTM1* on gene expression may partially explain the contradictory results from those case control studies which lack dietary assessment and which have or have not associated the *GSTM1* null genotype with enhanced risk of prostate cancer [44]–[47]. *GSTM1* enzyme activity catalyses both the formation and the cleavage of SF – glutathione conjugates [48]. We suggest that following transport into the plasma from enterocytes, *GSTM1* activity (originating either from hepatic cell turnover [49] or leakage from peripheral lymphocytes [50]) catalyses the cleavage of the SF-glutathione conjugate within the low glutathione environment of the plasma [51] to determine the extent of free SF that is available for protein modification, as discussed above, and which is not excreted via mercapturic acid metabolism (Figure 1). Thus low levels of SF, as would be expected from normal dietary consumption of broccoli, may lead to subtle changes in cell signalling, which, over time, result in profound changes in gene expression. In this manner, consuming one portion of broccoli per week if one is *GSTM1* positive, or more if one is *GSTM1* null [14], may contribute to a reduction in cancer risk.

In addition to the insight this study provides to the effect of broccoli consumption on gene expression, we consider that our study may have broader implications. First, we demonstrate that routine prostate needle biopsies can be used for global gene expression analyses in addition to histological assessment, and that it is possible to monitor changes in expression with time. It is notable that men within both dietary arms of the study had significant changes in the androgen receptor pathway. It is possible that these changes in androgen signalling are associated with aging and independent of diets, or they may have been induced by a common component of both the broccoli-rich and pea-rich diet. To our knowledge there is no data on the rate of change on androgen signalling in men of this age with HGPIN. This observation suggests further study is warranted. Analysis of the rate of change of gene expression of men diagnosed with either HGPIN or localized prostate cancer through sequential biopsies may provide reliable biomarkers to measure the likelihood of both carcinogenesis and progression to aggressive cancer, and complement histological examination of needle biopsies and measurement of plasma PSA levels. Secondly, stratification of global gene expression profiles by genotype has been informative, and this approach could be extended to other genes to dissect patterns of gene expression in prostate or other tissues. Lastly, it is conceivable that other dietary phytochemicals, such as polyphenolic derivatives, could also chemically interact with signalling peptides in the plasma, in a similar manner to the suggested mechanism of action of isothiocyanates.

In conclusion, we consider that our study has provided a mechanistic basis for the reduction in risk of prostate cancer through broccoli consumption, as suggested by epidemiological studies. Further studies with larger cohorts, combined with the assessment of clinical endpoints, are warranted.

## Supporting Information

Table S1 Primers and probes for genotype analysis.
(0.05 MB DOC)

Table S2 Relative expression of probes belonging to the TGFβ receptor pathway in *GSTM1* positive individuals compared with *GSTM1* nulls following six months broccoli-rich diet (P≤0.05).
(0.10 MB DOC)

Table S3 Relative expression of probes belonging to the EGF receptor pathway in *GSTM1* positive individuals compared with *GSTM1* nulls following six months broccoli-rich diet (P≤0.05).
(0.12 MB DOC)

Table S4 Change in expression of probes of the TGFβ receptor pathway in paired samples before and after a 12 month broccoli-rich diet.
(0.08 MB DOC)

Table S5 Change in expression of probes of the EGF receptor pathway in paired samples before and after a 12 month broccoli-rich diet.
(0.09 MB DOC)

Protocol S1 Trial Protocol.
(0.31 MB DOC)

Checklist S1 CONSORT Checklist.
(0.07 MB DOC)

Text S1 Approval from Norwich Research Ethics Committee.
(0.07 MB DOC)

Figure S1 CONSORT flow diagram.
(0.03 MB DOC)

Figure S2 Modification of TGFβ1 by SF and iodoacetamide.
(0.07 MB DOC)
