# Supplementary material for: Broccoli Consumption Interacts with GSTM1 to Perturb Oncogenic Signalling Pathways in the Prostate
Source: PLoS One. 2008 Jul 2;3(7):e2568. doi: 10.1371/journal.pone.0002568 (PMC2430620; doi:10.1371/journal.pone.0002568)
Supplement: Table S1 — Primers and probes for genotype analysis. (0.05 MB DOC) [file pone.0002568.s001.doc]

| Table S1. Primers and probes for genotype analysis. | | |
| --- | --- | --- |
| **Gene** | **Sequence** | **primer & probe (nM)** |
| GSTM1 - glutathione S-transferase M1 | |  |
| F | 5'- GGAGACAGAAGAGGAGAAGATTCG -3' | 500 |
| R | 5’- TGCCCAGCTGCATATGGTT -3’ | 500 |
| P | 5’- TCCATGGTCTGGTTCTCCAAAATGTCCA -3’ | 200 |
| Control gene (*BRCA1*) | |  |
| F | 5’- GTCTGCTTTTACATCTGAACCTCTGT -3’ | 500 |
| R | 5’- AGCCCTGAGCAGTCTTCAGAGA -3’ | 500 |
| P | 5’- ACTCTCACACCCAGATGCTGCTTCACCT -3’ | 200 |

Sequences and concentration of forward (F) and reverse (R) primers and fluorogenic probes (P) for the determination of GSTM1 gene deletion are shown. Probes were labelled with a 5’ reporter dye, FAM (6-carboxyfluorescein) and a 3’ quencher dye, TAMRA (6- carboxytetramethylthodamine). Triplicate reactions were carried out in a total volume of 25 μL/well consisting of Universal MasterMix, primers and probes and 50 ng DNA. Amplitaq Gold activation for 10 min at 95°C, followed by 40 cycles PCR of denaturation for 15 s at 95°C and annealing/extension for 1 min at 60°C.
