# Supplementary material for: Broccoli Consumption Interacts with GSTM1 to Perturb Oncogenic Signalling Pathways in the Prostate
Source: PLoS One. 2008 Jul 2;3(7):e2568. doi: 10.1371/journal.pone.0002568 (PMC2430620; doi:10.1371/journal.pone.0002568)
Supplement: Table S2 — Relative expression of probes belonging to the TGFβ receptor pathway in GSTM1 positive individuals compared with GSTM1 nulls following six months broccoli-rich diet (P≤0.05). (0.10 MB DOC) [file pone.0002568.s002.doc]

| **Table S2.** Relative expression of probes belonging to the TGFβ receptor pathway in *GSTM1* positive individuals compared with *GSTM1* nulls following six months broccoli-rich diet (*P*≤0.05). | | | | |
| --- | --- | --- | --- | --- |
| **probe set** | **Gene name** | **Accession** | **Relative expression** | ***P*-value** |
| 209956_s_at | calcium/calmodulin-dependent protein kinase (CaM kinase) II beta | U23460 | 1.83 | 0.009 |
| 208429_x_at | hepatocyte nuclear factor 4, alpha | NM_000457 | 1.72 | 0.025 |
| 238556_at | Calcium/calmodulin-dependent protein kinase (CaM kinase) II gamma | AW295338 | 1.62 | 0.033 |
| 217577_at | Exportin 1 (CRM1 homolog, yeast) | AW576871 | 1.58 | 0.010 |
| 201808_s_at | endoglin (Osler-Rendu-Weber syndrome 1) | BE732652 | 1.55 | 0.045 |
| 211087_x_at | mitogen-activated protein kinase 14 | Z25432 | 1.51 | 0.020 |
| 1554420_at | activating transcription factor 3 | AB078026 | 1.47 | 0.016 |
| 221282_x_at | runt-related transcription factor 2 | NM_004348 | 1.46 | 0.020 |
| 204843_s_at | protein kinase, cAMP-dependent, regulatory, type II, alpha | NM_004157 | 1.43 | 0.028 |
| 211235_s_at | estrogen receptor 1 | AF258450 | 1.4 | 0.048 |
| 229117_s_at | Jun D proto-oncogene | AI337326 | 1.38 | 0.042 |
| 231407_s_at | Forkhead box H1 | AI636647 | 1.38 | 0.008 |
| 212559_at | protein kinase, cAMP-dependent, regulatory, type I, beta | AU148827 | 1.37 | 0.033 |
| 38707_r_at | E2F transcription factor 4, p107/p130-binding | S75174 | 1.36 | 0.045 |
| 244858_at | TGFB-induced factor (TALE family homeobox) | BF507848 | 1.36 | 0.026 |
| 222374_at | beta-transducin repeat containing | AI186064 | 1.31 | 0.036 |
| 236669_at | (clone 33) macronuclear mRNA. | AI038054 | 1.31 | 0.024 |
| 235480_at | Mitogen-activated protein kinase kinase kinase 7 interacting protein 1 | AA063633 | 1.3 | 0.009 |
| 209415_at | fizzy/cell division cycle 20 related 1 (Drosophila) | AA905473 | 1.28 | 0.031 |
| 224176_s_at | axin 2 (conductin, axil) | AF205888 | 1.21 | 0.038 |
| 207105_s_at | phosphoinositide-3-kinase, regulatory subunit 2 (p85 beta) | NM_005027 | 1.19 | 0.036 |
| 202313_at | protein phosphatase 2 (formerly 2A), regulatory subunit B (PR 52), alpha isoform | NM_002717 | -1.19 | 0.029 |
| 213507_s_at | karyopherin (importin) beta 1 | BG249565 | -1.23 | 0.031 |
| 229355_at | Ubiquitin-conjugating enzyme E2D 3 (UBC4/5 homolog, yeast) | AU150386 | -1.23 | 0.049 |
| 206853_s_at | Mitogen-activated protein kinase kinase kinase 7 | AL121964 | -1.24 | 0.025 |
| 217880_at | Cell division cycle 27 | N21397 | -1.24 | 0.037 |
| 202215_s_at | nuclear transcription factor Y, gamma | NM_014223 | -1.25 | 0.021 |
| 205596_s_at | SMAD specific E3 ubiquitin protein ligase 2 | AY014180 | -1.25 | 0.025 |
| 202521_at | CCCTC-binding factor (zinc finger protein) | NM_006565 | -1.27 | 0.021 |
| 203075_at | SMAD, mothers against DPP homolog 2 (Drosophila) | AW151617 | -1.27 | 0.025 |
| 213044_at | Homo sapiens, Similar to Rho-associated, coiled-coil containing protein kinase 1, clone IMAGE:5269982, mRNA | N22548 | -1.27 | 0.045 |
| 226917_s_at | anaphase promoting complex subunit 4 | AA604393 | -1.28 | 0.017 |
| 231973_s_at | anaphase promoting complex subunit 1 | AK001223 | -1.28 | 0.044 |
| 203845_at | p300/CBP-associated factor | AV727449 | -1.29 | 0.034 |
| 201746_at | tumor protein p53 (Li-Fraumeni syndrome) | NM_000546 | -1.32 | 0.024 |
| 235926_at | Anaphase promoting complex subunit 5 | AI312527 | -1.33 | 0.037 |
| 202724_s_at | forkhead box O1A (rhabdomyosarcoma) | NM_002015 | -1.34 | 0.014 |
| 217862_at | protein inhibitor of activated STAT, 1 | N24868 | -1.34 | 0.012 |
| 201345_s_at | ubiquitin-conjugating enzyme E2D 2 (UBC4/5 homolog, yeast) | NM_003339 | -1.35 | 0.027 |
| 207614_s_at | cullin 1 | NM_003592 | -1.35 | 0.024 |
| 213579_s_at | E1A binding protein p300 | AI459462 | -1.36 | 0.040 |
| 212535_at | MADS box transcription enhancer factor 2, polypeptide A (myocyte enhancer factor 2A) | AA142929 | -1.39 | 0.030 |
| 217523_at | CD44 antigen (homing function and Indian blood group system) | AV700298 | -1.41 | 0.011 |
| 224894_at | Yes-associated protein 1, 65kDa | BF247906 | -1.42 | 0.046 |
| 222410_s_at | sorting nexin 6 | AF121856 | -1.43 | 0.043 |
| 202097_at | nucleoporin 153kDa | NM_005124 | -1.45 | 0.015 |
| 218127_at | nuclear transcription factor Y, beta | AI804118 | -1.45 | 0.034 |
| 224793_s_at | transforming growth factor, beta receptor I (activin A receptor type II-like kinase, 53kDa) | AA604375 | -1.47 | 0.038 |
| 209106_at | nuclear receptor coactivator 1 | BF576458 | -1.49 | 0.005 |
| 212240_s_at | phosphoinositide-3-kinase, regulatory subunit 1 (p85 alpha) | AI679268 | -1.51 | 0.013 |
| 231793_s_at | calcium/calmodulin-dependent protein kinase (CaM kinase) II delta | AA448956 | -1.55 | 0.042 |
| 204790_at | SMAD, mothers against DPP homolog 7 (Drosophila) | NM_005904 | -1.61 | 0.030 |
| 212154_at | syndecan 2 (heparan sulfate proteoglycan 1, cell surface-associated, fibroglycan) | AI380298 | -1.64 | 0.047 |
| 224851_at | cyclin-dependent kinase 6 | AW274756 | -1.7 | 0.034 |
| 204731_at | transforming growth factor, beta receptor III (betaglycan, 300kDa) | NM_003243 | -1.8 | 0.016 |
| 209199_s_at | MADS box transcription enhancer factor 2, polypeptide C (myocyte enhancer factor 2C) | N22468 | -1.81 | 0.023 |
| 203085_s_at | transforming growth factor, beta 1 (Camurati-Engelmann disease) | BC000125 | -2.12 | 0.011 |
| 224833_at | v-ets erythroblastosis virus E26 oncogene homolog 1 (avian) | BE218980 | -2.3 | 0.028 |
