# Supplementary material for: Broccoli Consumption Interacts with GSTM1 to Perturb Oncogenic Signalling Pathways in the Prostate
Source: PLoS One. 2008 Jul 2;3(7):e2568. doi: 10.1371/journal.pone.0002568 (PMC2430620; doi:10.1371/journal.pone.0002568)
Supplement: Table S3 — Relative expression of probes belonging to the EGF receptor pathway in GSTM1 positive individuals compared with GSTM1 nulls following six months broccoli-rich diet (P≤0.05). (0.12 MB DOC) [file pone.0002568.s003.doc]

| **Table S3.** Relative expression of probes belonging to the EGF receptor pathway in *GSTM1* positive individuals compared with *GSTM1* nulls following six months broccoli-rich diet (*P*≤0.05). | | | | |
| --- | --- | --- | --- | --- |
| **probe set** | **Gene name** | **Accession** | **Relative expression** | ***P*-value** |
| 204906_at | ribosomal protein S6 kinase, 90kDa, polypeptide 2 | BC002363 | 1.86 | 0.023 |
| 205810_s_at | Wiskott-Aldrich syndrome-like | NM_003941 | 1.8 | 0.017 |
| 218030_at | G protein-coupled receptor kinase interactor 1 | NM_014030 | 1.61 | 0.044 |
| 243482_at | Epidermal growth factor receptor pathway substrate 15-like 1 | BF512299 | 1.61 | 0.039 |
| 232218_at | gb:AI937687 /DB_XREF=gi:5676557 /DB_XREF=wp82d06.x1 | AI937687 | 1.6 | 0.014 |
| 1565080_at | v-src sarcoma (Schmidt-Ruppin A-2) viral oncogene homolog (avian) | AF272982 | 1.58 | 0.023 |
| 1565633_at | Catenin (cadherin-associated protein), delta 1 | AK024528 | 1.58 | 0.034 |
| 203514_at | mitogen-activated protein kinase kinase kinase 3 | BF971923 | 1.55 | 0.039 |
| 206607_at | Cas-Br-M (murine) ecotropic retroviral transforming sequence | NM_005188 | 1.51 | 0.015 |
| 211087_x_at | mitogen-activated protein kinase 14 | Z25432 | 1.51 | 0.020 |
| 209744_x_at | itchy homolog E3 ubiquitin protein ligase (mouse) | AB056663 | 1.5 | 0.027 |
| 217576_x_at | son of sevenless homolog 2 (Drosophila) | BF692958 | 1.5 | 0.044 |
| 234543_at | USP6 N-terminal like | AL050124 | 1.5 | 0.044 |
| 205157_s_at | keratin 17 | NM_000422 | 1.49 | 0.016 |
| 212777_at | son of sevenless homolog 1 (Drosophila) | L13857 | 1.46 | 0.039 |
| 205426_s_at | huntingtin interacting protein 1 | U79734 | 1.44 | 0.011 |
| 216551_x_at | phospholipase C, gamma 1 | AL110247 | 1.44 | 0.034 |
| 1568448_at | Ribosomal protein S6 kinase, 90kDa, polypeptide 3 | AJ012498 | 1.44 | 0.029 |
| 1558394_s_at | keratin 7 | BC042076 | 1.41 | 0.008 |
| 209410_s_at | growth factor receptor-bound protein 10 | AF000017 | 1.4 | 0.006 |
| 211550_at | epidermal growth factor receptor (erythroblastic leukemia viral (v-erb-b) oncogene homolog, avian) | AF125253 | 1.4 | 0.047 |
| 233471_at | protein tyrosine phosphatase, non-receptor type 5 (striatum-enriched) | U27831 | 1.4 | 0.048 |
| 202844_s_at | ralA binding protein 1 | AW025261 | 1.39 | 0.028 |
| 229117_s_at | Jun D proto-oncogene | AI337326 | 1.38 | 0.042 |
| 205170_at | signal transducer and activator of transcription 2, 113kDa | NM_005419 | 1.37 | 0.013 |
| 244858_at | TGFB-induced factor (TALE family homeobox) | BF507848 | 1.36 | 0.026 |
| 210825_s_at | prostatic binding protein | AF130103 | 1.32 | 0.030 |
| 240524_x_at | gb:BE466632 /DB_XREF=gi:9512407 /DB_XREF=hz23d06.x1 | BE466632 | 1.31 | 0.044 |
| 204756_at | mitogen-activated protein kinase kinase 5 | NM_002757 | 1.3 | 0.031 |
| 204039_at | CCAAT/enhancer binding protein (C/EBP), alpha | NM_004364 | 1.28 | 0.021 |
| 205868_s_at | protein tyrosine phosphatase, non-receptor type 11 (Noonan syndrome 1) | L07527 | 1.28 | 0.047 |
| 208156_x_at | epiplakin 1 | NM_031308 | 1.26 | 0.030 |
| 216439_at | tyrosine kinase, non-receptor, 2 | AK024904 | 1.26 | 0.048 |
| 207105_s_at | phosphoinositide-3-kinase, regulatory subunit 2 (p85 beta) | NM_005027 | 1.19 | 0.036 |
| 35617_at | mitogen-activated protein kinase 7 | U29725 | 1.19 | 0.046 |
| 213477_x_at | eukaryotic translation elongation factor 1 alpha 1 | AL515273 | 1.14 | 0.041 |
| 227520_at | Chromosome X open reading frame 15 | AI885312 | -1.16 | 0.040 |
| 200054_at | zinc finger protein 259 /// zinc finger protein 259 | NM_003904 | -1.22 | 0.046 |
| 201598_s_at | inositol polyphosphate phosphatase-like 1 | NM_001567 | -1.22 | 0.049 |
| 202677_at | RAS p21 protein activator (GTPase activating protein) 1 | NM_002890 | -1.26 | 0.032 |
| 203075_at | SMAD, mothers against DPP homolog 2 (Drosophila) | AW151617 | -1.27 | 0.025 |
| 218158_s_at | adaptor protein containing pH domain, PTB domain and leucine zipper motif 1 | NM_012096 | -1.28 | 0.009 |
| 203315_at | NCK adaptor protein 2 | BC000103 | -1.31 | 0.008 |
| 207196_s_at | TNFAIP3 interacting protein 1 | NM_006058 | -1.31 | 0.022 |
| 201667_at | gap junction protein, alpha 1, 43kDa (connexin 43) | NM_000165 | -1.32 | 0.010 |
| 212549_at | signal transducer and activator of transcription 5B | BE645861 | -1.32 | 0.036 |
| 242482_at | protein kinase, cAMP-dependent, regulatory, type I, alpha (tissue specific extinguisher 1) | AI682905 | -1.32 | 0.032 |
| 202724_s_at | forkhead box O1A (rhabdomyosarcoma) | NM_002015 | -1.34 | 0.014 |
| 212629_s_at | protein kinase N2 | AI633689 | -1.34 | 0.009 |
| 202777_at | soc-2 suppressor of clear homolog (C. elegans) | NM_007373 | -1.35 | 0.016 |
| 217886_at | epidermal growth factor receptor pathway substrate 15 | BF213575 | -1.36 | 0.026 |
| 225225_at | Homo sapiens, clone IMAGE:5274897, mRNA | BF791544 | -1.37 | 0.021 |
| 214853_s_at | SHC (Src homology 2 domain containing) transforming protein 1 | AI091079 | -1.39 | 0.036 |
| 226441_at | Mitogen-activated protein kinase kinase kinase 2 | AA045204 | -1.4 | 0.002 |
| 1562031_at | Janus kinase 2 (a protein tyrosine kinase) | BC043187 | -1.41 | 0.030 |
| 224657_at | Gene 33/Mig-6 (MIG-6) | AL034417 | -1.42 | 0.024 |
| 212180_at | v-crk sarcoma virus CT10 oncogene homolog (avian)-like | AK000311 | -1.43 | 0.007 |
| 39313_at | WNK lysine deficient protein kinase 1 | AB002342 | -1.43 | 0.006 |
| 215075_s_at | growth factor receptor-bound protein 2 | L29511 | -1.49 | 0.018 |
| 221039_s_at | development and differentiation enhancing factor 1 | NM_018482 | -1.49 | 0.037 |
| 204725_s_at | NCK adaptor protein 1 | NM_006153 | -1.5 | 0.038 |
| 209682_at | Cas-Br-M (murine) ecotropic retroviral transforming sequence b | U26710 | -1.5 | 0.013 |
| 225927_at | mitogen-activated protein kinase kinase kinase 1 | AA541479 | -1.5 | 0.017 |
| 212240_s_at | phosphoinositide-3-kinase, regulatory subunit 1 (p85 alpha) | AI679268 | -1.51 | 0.013 |
| 222103_at | Activating transcription factor 1 | AI434345 | -1.53 | 0.008 |
| 1552263_at | mitogen-activated protein kinase 1 | NM_138957 | -1.54 | 0.015 |
| 203324_s_at | caveolin 2 | NM_001233 | -1.55 | 0.030 |
| 202743_at | phosphoinositide-3-kinase, regulatory subunit 3 (p55, gamma) | BE622627 | -1.61 | 0.003 |
| 229114_at | GRB2-associated binding protein 1 | AW237741 | -1.62 | 0.010 |
| 201648_at | Janus kinase 1 (a protein tyrosine kinase) | AL039831 | -1.66 | 0.012 |
| 202609_at | epidermal growth factor receptor pathway substrate 8 | NM_004447 | -1.78 | 0.036 |
| 212501_at | CCAAT/enhancer binding protein (C/EBP), beta | AL564683 | -1.78 | 0.034 |
| 202006_at | protein tyrosine phosphatase, non-receptor type 12 | NM_002835 | -1.84 | 0.004 |
| 238563_at | Abl-interactor 1 | AV762916 | -1.99 | 0.010 |
| 202430_s_at | phospholipid scramblase 1 | NM_021105 | -2.48 | 0.009 |
| 200887_s_at | signal transducer and activator of transcription 1, 91kDa | NM_007315 | -3.14 | 0.014 |
