# Supplementary material for: Broccoli Consumption Interacts with GSTM1 to Perturb Oncogenic Signalling Pathways in the Prostate
Source: PLoS One. 2008 Jul 2;3(7):e2568. doi: 10.1371/journal.pone.0002568 (PMC2430620; doi:10.1371/journal.pone.0002568)
Supplement: Table S4 — Change in expression of probes of the TGFβ receptor pathway in paired samples before and after a 12 month broccoli-rich diet. (0.08 MB DOC) [file pone.0002568.s004.doc]

| **Table S4.** Change in expression of probes of the TGFβ receptor pathway in paired samples before and after a 12 month broccoli-rich diet. | | | | |
| --- | --- | --- | --- | --- |
| **probe set** | **Gene name** | **Accession** | **Fold** | **paired *P*-value** |
| 243046_at | Ubiquitin-conjugating enzyme E2D 3 (UBC4/5 homolog, yeast) | BF679700 | 2.7 | 0.035 |
| 204131_s_at | forkhead box O3A | N25732 | 2.15 | 0.007 |
| 203065_s_at | caveolin 1, caveolae protein, 22kDa | NM_001753 | 2.01 | 0.037 |
| 215604_x_at | Ubiquitin-conjugating enzyme E2D 2 (UBC4/5 homolog, yeast) | AK023783 | 1.78 | 0.032 |
| 1570507_at | catenin (cadherin-associated protein), beta 1, 88kDa | BC020736 | 1.55 | 0.037 |
| 234125_at | Erbb2 interacting protein | AL137318 | 1.53 | 0.037 |
| 204731_at | transforming growth factor, beta receptor III (betaglycan, 300kDa) | NM_003243 | 1.49 | 0.039 |
| 207658_s_at | forkhead box G1B/G1A | NM_004471 | 1.49 | 0.045 |
| 218508_at | decapping enzyme | NM_018403 | 1.48 | 0.027 |
| 230749_s_at | calcium/calmodulin-dependent protein kinase (CaM kinase) II delta | AI829910 | 1.48 | 0.037 |
| 1559436_x_at | Arrestin, beta 2 | AL832061 | 1.48 | 0.037 |
| 212669_at | calcium/calmodulin-dependent protein kinase (CaM kinase) II gamma | AI093569 | 1.47 | 0.016 |
| 225000_at | Protein kinase, cAMP-dependent, regulatory, type II, alpha | BF246131 | 1.46 | 0.023 |
| 241435_at | V-ets erythroblastosis virus E26 oncogene homolog 1 (avian) | AA702930 | 1.41 | 0.029 |
| 244012_x_at | Exportin 1 (CRM1 homolog, yeast) | AI761130 | 1.3 | 0.048 |
| 200090_at | farnesyltransferase, CAAX box, alpha | BG168896 | 1.24 | 0.016 |
| 224754_at | Sp1 transcription factor | BG431266 | 1.23 | 0.038 |
| 212239_at | phosphoinositide-3-kinase, regulatory subunit 1 (p85 alpha) | AI680192 | -1.24 | 0.039 |
| 207334_s_at | transforming growth factor, beta receptor II (70/80kDa) | NM_003242 | -1.24 | 0.040 |
| 200711_s_at | S-phase kinase-associated protein 1A (p19A) | NM_003197 | -1.25 | 0.019 |
| 212758_s_at | transcription factor 8 (represses interleukin 2 expression) | AI373166 | -1.27 | 0.045 |
| 200870_at | serine/threonine kinase receptor associated protein | NM_007178 | -1.28 | 0.022 |
| 207143_at | cyclin-dependent kinase 6 | NM_001259 | -1.28 | 0.045 |
| 218127_at | nuclear transcription factor Y, beta | AI804118 | -1.3 | 0.032 |
| 211764_s_at | ubiquitin-conjugating enzyme E2D 1 (UBC4/5 homolog, yeast) | BC005980 | -1.43 | 0.018 |
| 211797_s_at | nuclear transcription factor Y, gamma | U62296 | -1.43 | 0.025 |
| 211537_x_at | mitogen-activated protein kinase kinase kinase 7 | AF218074 | -1.44 | 0.011 |
| 211540_s_at | retinoblastoma 1 (including osteosarcoma) | M19701 | -1.45 | 0.045 |
| 1555003_at | retinoblastoma-like 1 (p107) | BC032247 | -1.45 | 0.050 |
| 239271_at | SMAD, mothers against DPP homolog 2 (Drosophila) | AV698619 | -1.47 | 0.029 |
| 203313_s_at | TGFB-induced factor (TALE family homeobox) | NM_003244 | -1.47 | 0.036 |
| 212652_s_at | sorting nexin 4 | AA524345 | -1.48 | 0.025 |
| 212332_at | retinoblastoma-like 2 (p130) | BF110947 | -1.52 | 0.012 |
| 224895_at | Yes-associated protein 1, 65kDa | AA557632 | -1.58 | 0.009 |
| 208711_s_at | cyclin D1 (PRAD1: parathyroid adenomatosis 1) | BC000076 | -1.68 | 0.032 |
| 209105_at | nuclear receptor coactivator 1 | AI672428 | -1.7 | 0.042 |
| 210186_s_at | FK506 binding protein 1A, 12kDa | BC005147 | -1.89 | 0.009 |
| 201473_at | jun B proto-oncogene | NM_002229 | -2.09 | 0.045 |
| 209909_s_at | transforming growth factor, beta 2 | M19154 | -2.13 | 0.004 |
