# Supplementary material for: Broccoli Consumption Interacts with GSTM1 to Perturb Oncogenic Signalling Pathways in the Prostate
Source: PLoS One. 2008 Jul 2;3(7):e2568. doi: 10.1371/journal.pone.0002568 (PMC2430620; doi:10.1371/journal.pone.0002568)
Supplement: Table S5 — Change in expression of probes of the EGF receptor pathway in paired samples before and after a 12 month broccoli-rich diet. (0.09 MB DOC) [file pone.0002568.s005.doc]

| **Table S5.** Change in expression of probes of the EGF receptor pathway in paired samples before and after a 12 month broccoli-rich diet. | | | | |
| --- | --- | --- | --- | --- |
| **probe set** | **Gene name** | **Accession** | **Fold** | **paired *P*-value** |
| 225332_at | Keratin associated protein 4-7 | BF674064 | 2.04 | 0.008 |
| 203065_s_at | caveolin 1, caveolae protein, 22kDa | NM_001753 | 2.01 | 0.037 |
| 221039_s_at | development and differentiation enhancing factor 1 | NM_018482 | 1.73 | 0.006 |
| 202224_at | v-crk sarcoma virus CT10 oncogene homolog (avian) | BF304695 | 1.71 | 0.019 |
| 204010_s_at | v-Ki-ras2 Kirsten rat sarcoma viral oncogene homolog | NM_004985 | 1.59 | 0.027 |
| 208991_at | signal transducer and activator of transcription 3 (acute-phase response factor) | AA634272 | 1.59 | 0.004 |
| 203843_at | ribosomal protein S6 kinase, 90kDa, polypeptide 3 | AA906056 | 1.56 | 0.047 |
| 216206_x_at | mitogen-activated protein kinase kinase 7 | BC005365 | 1.54 | 0.031 |
| 211565_at | SH3-domain GRB2-like 3 | AF036272 | 1.51 | 0.033 |
| 217705_at | protein kinase D1 | AW085172 | 1.51 | 0.039 |
| 225636_at | signal transducer and activator of transcription 2, 113kDa | H98105 | 1.49 | 0.027 |
| 208641_s_at | ras-related C3 botulinum toxin substrate 1 (rho family, small GTP binding protein Rac1) | BC004247 | 1.39 | 0.043 |
| 213426_s_at | caveolin 2 | AA150110 | 1.33 | 0.035 |
| 232442_at | Breast cancer anti-estrogen resistance 1 | AU147442 | 1.32 | 0.022 |
| 200826_at | small nuclear ribonucleoprotein D2 polypeptide 16.5kDa | NM_004597 | 1.27 | 0.041 |
| 201190_s_at | phosphatidylinositol transfer protein, alpha | H15647 | 1.27 | 0.033 |
| 224754_at | Sp1 transcription factor | BG431266 | 1.23 | 0.038 |
| 202178_at | protein kinase C, zeta | NM_002744 | 1.21 | 0.049 |
| 229617_x_at | adaptor-related protein complex 2, alpha 1 subunit | AA729495 | 1.2 | 0.024 |
| 212239_at | phosphoinositide-3-kinase, regulatory subunit 1 (p85 alpha) | AI680192 | -1.24 | 0.039 |
| 224999_at | Epidermal growth factor receptor (erythroblastic leukemia viral (v-erb-b) oncogene homolog, avian) | BE878463 | -1.27 | 0.049 |
| 206219_s_at | vav 1 oncogene | NM_005428 | -1.31 | 0.027 |
| 202647_s_at | neuroblastoma RAS viral (v-ras) oncogene homolog | NM_002524 | -1.34 | 0.036 |
| 205426_s_at | huntingtin interacting protein 1 | U79734 | -1.39 | 0.041 |
| 209896_s_at | protein tyrosine phosphatase, non-receptor type 11 (Noonan syndrome 1) | AF119855 | -1.4 | 0.018 |
| 204633_s_at | ribosomal protein S6 kinase, 90kDa, polypeptide 5 | AF074393 | -1.44 | 0.049 |
| 229010_at | Cas-Br-M (murine) ecotropic retroviral transforming sequence | AI807026 | -1.44 | 0.047 |
| 202777_at | soc-2 suppressor of clear homolog (C. elegans) | NM_007373 | -1.47 | 0.020 |
| 203313_s_at | TGFB-induced factor (TALE family homeobox) | NM_003244 | -1.47 | 0.036 |
| 222538_s_at | adaptor protein containing pH domain, PTB domain and leucine zipper motif 1 | AW467472 | -1.47 | 0.012 |
| 239271_at | SMAD, mothers against DPP homolog 2 (Drosophila) | AV698619 | -1.47 | 0.029 |
| 204039_at | CCAAT/enhancer binding protein (C/EBP), alpha | NM_004364 | -1.48 | 0.023 |
| 213324_at | v-src sarcoma (Schmidt-Ruppin A-2) viral oncogene homolog (avian) | AK024281 | -1.48 | 0.027 |
| 201096_s_at | ADP-ribosylation factor 4 | AL537042 | -1.5 | 0.047 |
| 221695_s_at | mitogen-activated protein kinase kinase kinase 2 | AF239798 | -1.52 | 0.007 |
| 225135_at | SIN3 homolog A, transcription regulator (yeast) | AI433017 | -1.6 | 0.008 |
| 205809_s_at | Wiskott-Aldrich syndrome-like | BE504979 | -1.64 | 0.006 |
| 209677_at | protein kinase C, iota | L18964 | -1.71 | 0.039 |
| 200604_s_at | protein kinase, cAMP-dependent, regulatory, type I, alpha (tissue specific extinguisher 1) | M18468 | -1.72 | 0.003 |
| 217644_s_at | son of sevenless homolog 2 (Drosophila) | AI276593 | -1.72 | 0.014 |
