# Supplementary material for: Broccoli Consumption Interacts with GSTM1 to Perturb Oncogenic Signalling Pathways in the Prostate
Source: PLoS One. 2008 Jul 2;3(7):e2568. doi: 10.1371/journal.pone.0002568 (PMC2430620; doi:10.1371/journal.pone.0002568)
Supplement: Protocol S1 — Trial Protocol. (0.31 MB DOC) [file pone.0002568.s006.doc]

**Text S1.** Study protocol

### A HUMAN INTERVENTION TRIAL STUDYING

### GENE EXPRESSION IN HIGH-GRADE

### PROSTATIC INTRAEPITHELIAL NEOPLASIA

### FOLLOWING CONSUMPTION OF BROCCOLI OR PEAS

**Short Title: Broccoli, Peas and PIN**

**PROTOCOL**

**Version 9: 19^th^ April 2006**

**COLLABORATIVE RESEARCH BETWEEN**


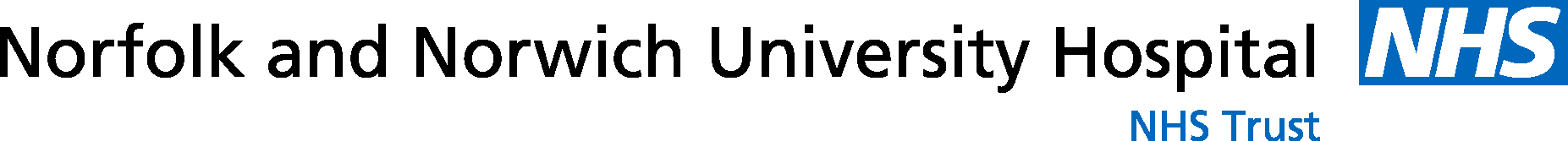


AND


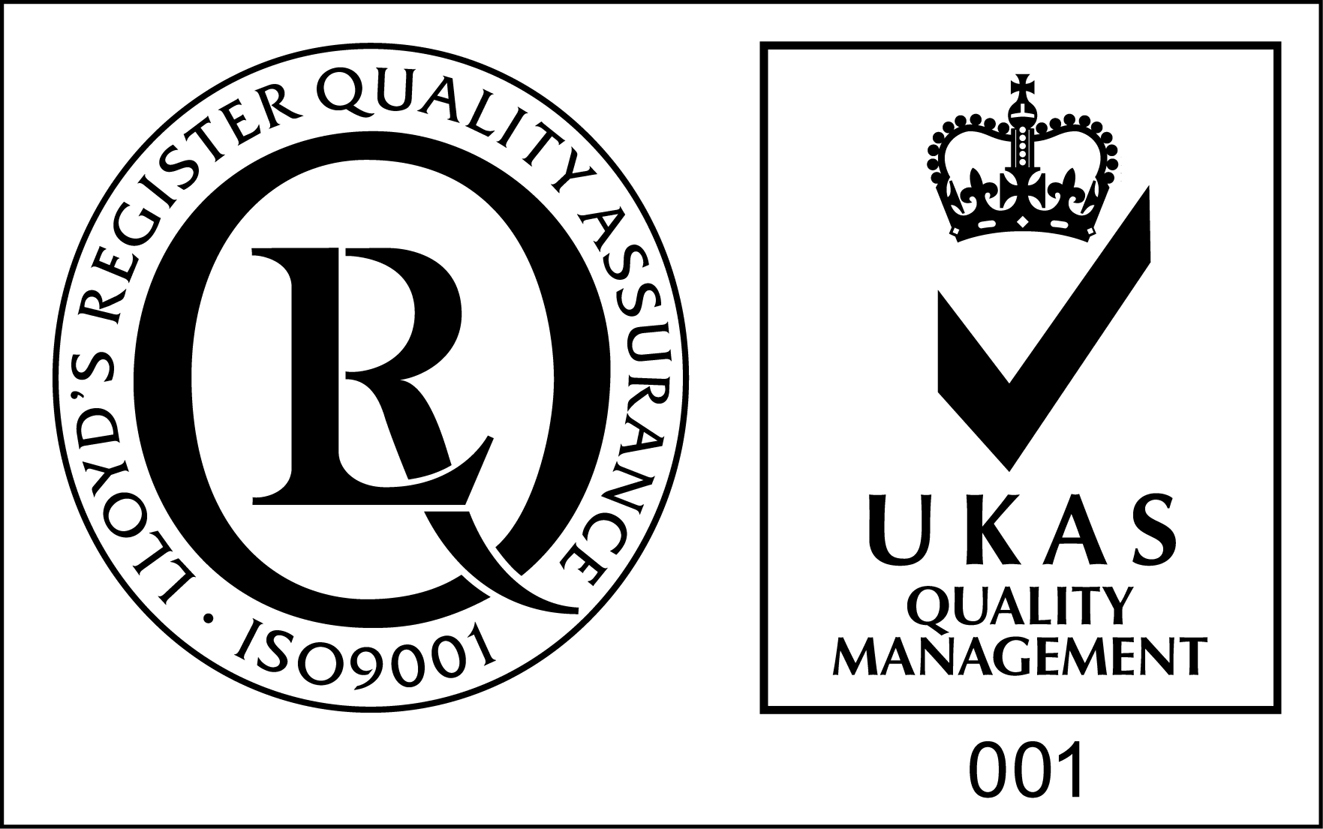


***Institute of Food Research***

**BACKGROUND**

Epidemiological evidence suggests that consumption of cruciferous vegetables, such as broccoli, is associated with a reduced risk of many types of cancers, Kristal (2002). More recently, this association has been linked with a possible protection against cancer of the prostate, Giovannucci *et al* (2003). The mechanism of chemoprevention is as yet unknown. However, the health benefits have been attributed to a major class of phytochemicals found in cruciferous vegetables known as glucosinolates.

References to the genus *Brassica* date back to ancient civilisation when its species were noted for their pungency and medicinal application, Fenwick *et al* (1983). The pungent, bitter taste can be attributed to the breakdown products of glucosinolates. When cruciferous vegetables are eaten, glucosinolates hydrolyse during the processes of chopping, chewing and ingestion, to form isothiocyanates and indoles. Broccoli contains various glucosinolates, but the most predominant are glucoraphanin, a precursor of sulforaphane [4-methylsulfinylbutyl ITC] and a potent anti-cancer compound in *in vitro* and animal studies, and glucoiberin (a precursor of the less bioactive ITC iberin [3-methylsulfinylpropyl ITC]), Mithen *et al* (2000).

*Mechanisms of Chemoprevention*

There is evidence that sulforaphane (SF) exerts an anti-carcinogenic effect by up-regulating various anti-carcinogenic enzymes involved in the detoxification and excretion of carcinogens. ITCs also down-regulate potentially pro-carcinogenic enzymes, such as cytochrome P450 enzymes, and enzymes involved in inflammatory responses that are also thought to be associated with cancer progression, Verhoeven *et al* (1997). Many recent studies are focusing on the involvement of SF in cell signal transduction pathways to elucidate its role in chemoprevention, Surh (2003).

The health-beneficial effects of ITCs have been investigated for many years; early work used pure compounds in animal studies and there is a substantial body of evidence that ITCs are effective inhibitors of carcinogenesis in a variety of animal models, Hecht (2000). Further evidence using cell line models has shown SF to up regulate the expression of genes involved in cell cycle suppression. This results in the suppression of growth and multiplication in human cancer cells originating from the liver, colon and prostate in vitro, Hintze et al (2003), Gamet-Payrastre et al (2000), Chiao et al (2002).

*Enhancing the Glucosinolate Content of Broccoli*

As the potential anticarcinogenic activity of broccoli has been associated with the presence of SF, a conventional breeding programme was set up at the John Innes Centre, Norwich, to selectively enhance the level of SF in broccoli. There is a wild cultivar of broccoli, originating from mountain tops in Sicily, which contains naturally high levels of glucosinolate. This cultivar was crossbred with a regular cultivar to produce a hybrid with increased levels of anticarcinogenic glucosinolate. This cultivar was developed specifically for use in human clinical intervention trials to allow further understanding of the role of ITCs in chemoprevention, Mithen *et al* (2003).

There are very few human intervention studies investigating the physiological significance of the action of glucosinolates on human health. Thus there is a great need to study pharmacokinetics, biomarkers of exposure and biomarkers of effect, in living people, following consumption of broccoli. Only then can we begin to interpret and understand the complexity of the role of broccoli in chemoprevention.

A recent human study was funded by the Biological and Biotechnological Sciences Research Council (BBSRC) and Nottingham University and approved by Queen’s Medical Centre Nottingham University Hospital NHS Trust Research Ethics Committee. Currently in the process of publication, this study examined the effects of acute exposure to ITCs in blood, urine and gastric tissue after consumption of broccoli enriched with glucosinolate. The study addressed methodological problems of measuring ITC metabolites in plasma, the effects of Glutathione-S-Transferase (GST) genotype on ITC exposure and changes in gene expression in the gastric mucosa of volunteers following consumption of enriched and standard broccoli cultivars. This is the same broccoli that will be used in the study proposed here.

*Genotype and Chemoprevention*

Several epidemiological studies suggest a further protection against cancer amongst individuals who have a deletion of certain genes, such as GSTs. This gene family convert reactive electrophiles, by conjugation with glutathione, to compounds that can be excreted from the body more easily. Approximately 40% of the population have a homologous deletion of the GSTM1 gene resulting in a null genotype. There is evidence that these individuals may have a higher protection by ITCs against certain cancers. We wish to explore this association further by genotyping the study population for members of the GST gene family.

*Justification for Pilot Intervention Trial*

In order to elucidate the mechanisms of chemoprevention of ITCs it is necessary to conduct a long term human intervention of broccoli to compare chronic exposure to ITC-enriched broccoli with the results of the acute clinical trial conducted in Nottingham. We wish to target men who have been previously diagnosed with high-grade Prostatic Intraepithelial Neoplasia (PIN) at the Norfolk and Norwich University Hospital by histopathologists with a special interest in prostate diseases. These men are at high risk for developing invasive prostate cancer (85% by year 5) and undergo routine prostate needle biopsies at 6 monthly intervals post diagnosis. The samples required for the study are thus part of normal diagnostic procedure and would provide very little additional inconvenience for the volunteers. The men would normally receive no medication unless cancer is detected and as such we anticipate a high compliance with the dietary intervention.

Prostate biopsy tissue will be taken before and after intervention of either broccoli or peas. We are primarily interested in the effect of broccoli on gene expression. However, peas are a good control because they cause the same change to the volunteers’ dietary regimen as adding broccoli and because they contain negligible amounts of glucosinolates. There is epidemiological evidence that peas may also be beneficial in reducing prostate cancer risk, but by different pathways to SF mediated action, Key *et al* (1997).

The study will use changes in gene expression as the main biomarker of effect. RNA will be extracted from the tissue and hybridised onto Affymetrix arrays. We will use validated software to create a gene expression profile of functional clusters of genes that are perturbed by the broccoli intervention. Subsequent analysis of selected genes by RT-PCR will confirm any significant changes in gene expression.

**OBJECTIVES**

1. To compare changes in gene expression, following a 12-month intervention of either broccoli or peas in subjects with high-grade PIN.
2. To assess changes in Prostate Specific Antigen (PSA) levels in response to a 12-month intervention of broccoli or peas in the study population.
3. To determine the genotype of individuals for key polymorphic genes and relate them to observed changes in gene expression.

**HYPOTHESIS**

Long-term consumption of broccoli results in changes in gene expression that may contribute to a reduction in cancer development and progression.

**STUDY DESIGN**

The study will be led by Professor Richard Mithen and Ms Amy Gasper at the Institute of Food Research, Norwich, in collaboration with Mr Robert Mills and Professor Richard Ball at the Urology and Histopathology departments of the Norfolk and Norwich University Hospital (NNUH) NHS Trust. A flow chart of the overall study is given in figure 1.

This is a parallel, dietary intervention studying male patients with high-grade PIN. Patients will be matched by genotype and diet into two dietary groups: (i) consuming 400g ITC-enriched broccoli per week (n=20) or (ii) consuming 400g peas per week (n=20). The study will involve dietary monitoring and collection of blood, urine and prostatic biopsy samples in a 12-month dietary intervention period. The prostate biopsies and blood will be collected during routine endoscopies as part of normal diagnostic procedure for this group of patients. The urine samples will be additional to routine procedure. It is anticipated that the drop out rate for this study will be 40% due to the progression of high-grade PIN to prostate cancer within the 12-month study period.

*Figure 1. Study Design.*

**ROLLING RECRUITMENT**

Male volunteers aged 30-70 years recruited by consultant urologist at NNUH. Information sheets and letters of invitation given to volunteers

**INFORMATION EXCHANGE**

Interested volunteers will be referred to study scientist at IFR and invited to attend an interview at the Human Nutrition Unit for initial information exchange

**CONSENT AND BASELINE BLOOD SAMPLE**

Interested volunteers will give informed, signed consent at the HNU. Volunteers will complete a health questionnaire and provide a baseline blood sample

#### SAMPLES

See figure 2

**DIETARY ASSESSMENT 1**

Recruited volunteers will complete a 7-day food intake diary and receive instructions on how to complete a dietary score sheet. Volunteers will be given a demonstration on how to cook the vegetable portions in a steamer.

**CLINIC APPOINTMENT 1**

Sample collection

#### SAMPLES

See figure 2

#### Group Allocation

**BROCCOLI GROUP**

Eat 4x100g portions of broccoli per week for 12 months

**PEAS GROUP**

Eat 4x100g portions of peas per week for 12 months

**DIETARY INTERVENTION PERIOD 1 (6 MONTHS)**

Fill in dietary score sheet

**DIETARY ASSESSMENT 2**

7 day food intake diary (rolling week basis) at 3-4 months

##

**CLINIC APPOINTMENT 2**

Sample collection

#### SAMPLES

See figure 2

**DIETARY INTERVENTION PERIOD 2 (6 MONTHS)**

Fill in dietary score sheet

**DIETARY ASSESSMENT 3**

7 day food intake diary (rolling week basis) at 10-11 months

**CLINIC APPOINTMENT 3**

Sample Collection

#### SAMPLES

See figure 2

**Recruitment Policy**

Patients with high-grade PIN, aged 30-70 years will be identified and recruited by Robert Mills, consultant urologist at the NNUH. Following a verbal expression of interest in the study, Mr Mills will send a letter (annex 1) with a volunteer information sheet (annex 2) inviting potential volunteers to take part in the study. Volunteers will return a reply slip to IFR, contact details of the study scientist (Ms Amy Gasper) will be provided to all potential volunteers.

Volunteers will be matched by genotype into two dietary groups: (i) consuming 400g ITC-enriched broccoli per week (n=20) or (ii) consuming 400g peas per week (n=20). Volunteer recruitment will continue until a total of 40 men have completed the trial. It is envisaged that 17 patients will be recruited per group and that the drop out rate for this study will be 40% due to the progression from high-grade PIN to prostate cancer within the 12-month study period. Interested volunteers will be invited to an information exchange at the Human Nutrition Unit (HNU) at IFR where the study scientist will explain details of the trial and encourage the volunteers to ask any questions. During this consideration period the volunteers will not be contacted.

*Inclusion criteria:*

- Male
- Diagnosed with high-grade PIN
- Aged between 30 and 70 years
- Smokers and non-smokers

*Exclusion criteria:*

- Undergoing chemopreventative therapy
- Receiving testosterone replacement medicines
- Prescribed 5α reductase inhibiters
- Active infection requiring treatment
- BMI <18.5 or >35
- Unable to give informed consent to take part
- Diagnosed with diabetes

**Consent and Baseline Blood Sample**

All volunteers responding positively following the period of consideration will be invited to the HNU for a blood sample. On arrival at the HNU, the study scientist will go through the consent form with the volunteer (annex 3) and encourage any questions they may have at this stage; after which the volunteer will sign the consent form agreeing to participate in the study. Copies of the consent form will be kept in the study records, the patient hospital notes and a copy given to the volunteer to keep. GPs will be informed by letter (annex 4) of their patients’ participation in the study.

A Research Nurse at the HNU, IFR will complete a basic health questionnaire with the volunteer. This includes measurements of blood pressure, height and weight, BMI and a medication declaration form agreeing to inform the study scientist of any medication they may have to take (annex 5). Volunteers will be excluded if they are found to have depressed or elevated blood pressure (<90/50 or >160/100) or BMI <18.5 or >35. A 10ml (2 teaspoons) blood sample will be taken by venepuncture by a qualified Research Nurse. This blood sample will be required in order to extract DNA from whole blood to determine genotypes for GSTM1, GSTT1 and GSTPi genes.

Once recruited onto the study, volunteers will be assigned a code, only the study scientists approved by the Ethics Committee being able to link codes to volunteers. Samples to be used for determining GST genotypes will be double encrypted so that individuals cannot be linked to the resulting genotype. All personal information will be kept confidential and known only to the Chief and Principle Investigators, HNU Research Nurses and Medical Advisor and the volunteer’s GP.

**Dietary Assessment**

We wish to measure habitual diet in both groups since the control group may consume a high amount of cruciferous vegetables as part of their normal diet. We also want to investigate confounding factors of other nutrient groups such as energy intake and dietary fibre in relation to baseline gene expression. This will be measured using the IFR standard 7-day food diary of estimated intake (annex 6). Volunteers will be asked to include use of any dietary supplements or herbal treatments in the diary.

Volunteers will be instructed to record their intake of food, beverages and supplements consumed over a 7-day period (incorporating 7 different days, including weekends) using household measures as an indication of portion size. Details of how to complete the diary are given in annex 6 along with an example diary. Halfway through and towards the end of the 12-month dietary intervention, 7-day food intake diaries will again be completed. The food intake diaries will be analysed using ‘Diet Cruncher’ and UK food composition tables.

**Compliance**

Volunteers will be asked to fill in a dietary record sheet during the 12-month intervention period, recording a tick each time they eat their vegetable portion (annex 7). This is intended to aid compliance of the dietary interventions and also to be used in comparison with the 7-day diet diary. Volunteers will be telephoned fortnightly by a member of the study team to monitor progress and the vegetable record sheet will be collected from the volunteer by a member of the study team to coincide with the delivery of frozen vegetables.

**Sample Collection**

Biopsy samples will be collected from the NNUH tissue bank and taken to IFR for analysis. Blood samples for PSA will be taken at the Urology clinic prior to the TRUS examination. Urine samples will be collected at the Urology clinic by a study scientist. Blood samples for genotyping will be collected at HNU. The plan for sample collection is detailed in figure 2.

*Blood Samples*

A 10ml blood sample will be collected at baseline for DNA extraction to determine genotype. DNA will be extracted at IFR. Further 5ml blood samples will be collected by venepuncture during the visits to the Urology Clinic (mid-way and post intervention) by a qualified nurse or phlebotomist. The samples will be analysed in the Biochemistry department at the NNUH for prostate specific antigen levels (PSA).

*Urine Samples*

At the mid-way time point, one 24 hour urine collection will be required from volunteers in each group following consumption of one of their vegetable meals. Volunteers will be provided with a labelled container (plus 1g ascorbic acid as a stabilising agent) in which to collect urine. They will be required to collect total urine volume immediately after consumption of a meal containing the peas or broccoli for 24 hours. The containers of urine will be collected by a study scientist at the urology clinic at the 6 month time point.

This 24 hour urine sample will provide qualitative evidence that the volunteers are receiving systemic exposure to ITCs because from previous investigations we know that if ITCs are present in the urine, they must also have been present in the plasma. Furthermore, this will provide useful information about the relative range of ITC excretion, absorption and metabolism in each of the volunteers. This will be vital for interpretation of the gene expression data. In addition the urine analysis will also confirm that the volunteers are cooking the broccoli correctly.

*Prostatic Biopsy Samples*

Volunteers will be asked to exclude cruciferous vegetables from their diet 48 hours prior to each visit to the clinic to avoid acute effects. The types of vegetables to avoid will be indicated in the dietary diaries. Biopsies of the prostate will be taken before, during and after the 12-month dietary intervention. The collection of all biopsy samples will occur at the urology clinic at the NNUH during routine Trans Rectal Ultrasound (TRUS) guided endoscopies. Thus, the samples required for the study will be collected at the same time as samples required as part of normal medical care. All patients will receive an explanation of the TRUS procedure by their consultant prior to the examination and a standard information sheet (annex 8).

The consultant will examine the prostate with an ultrasound probe. This is inserted into the rectum and images on a screen guide the sampling needles into the prostate. A small amount of tissue (10-20mg) is removed from the gland with the needles. The procedure takes approximately 30 minutes. As part of normal clinical care eight prostatic biopsy samples are collected for histology, in addition, up to six biopsy samples will be collected for the broccoli, peas and PIN study. The consultant will note from which lobe (right or left) the samples were taken.

Samples will be immersed in RNA later at the point of collection and subsequently snap frozen in liquid nitrogen before being transported to the tissue bank at the NNUH. A member of the study team will collect the samples from the tissue bank and transport to IFR for analysis. Tissue bank information sheets will be read (annex 10) and tissue bank consent forms (annex 9) signed by all volunteers prior to the TRUS procedure and copies will accompany all tissue transported to IFR.

*Progression to Prostate Cancer*

It is estimated that 30-40% of the study population may develop prostate cancer during the 12-month intervention. Volunteers diagnosed with cancer will be given appropriate medical counselling and advised to withdraw from the study by letter (annex 11). All non-medical members of the study team will be guided by the clinicians as to the most appropriate course of action for these individuals.

**Recruitment**

**Consent, Baseline Blood Sample**

10ml blood

**Dietary Assessment 1**

**Blood**

Genotype for GST family (10ml)

**Sample Collection 1**

Up to 6 prostatic biopsies

**Biopsy**

RNA expression (up to 6 cores)

**Intervention Period 1**

Distribution of broccoli and peas to volunteers

Compliance phone calls

**Urine**

ITCs and metabolites

1x24 hour collection

**Sample Collection 2**

Up to 6 prostatic biopsies

5ml blood

1x24 hour urine collection

**Intervention Period 2**

Distribution of broccoli and peas to volunteers

Compliance phone calls

**Sample Collection 3**

Up to 6 prostatic biopsies

5ml blood

**SAMPLING**

**Blood**

PSA (5ml)

**Biopsy**

RNA expression (up to 6 cores)

**Biopsy**

RNA expression (up to 6 cores)

**Blood**

PSA (5ml)

**STUDY DESIGN**

**Dietary Assessment 2**

**Dietary Assessment 3**

**Information Exchange**

**Blood**

PSA (5ml)

*Figure 2 Plan of sample collection*

**Dietary Intervention**

After giving fully informed, written consent, the volunteers will be matched by genotype and cruciferous vegetable consumption. The diet diary will be used to define high (> 3 portions per week) or low (< 1 portion a week) habitual consumers of cruciferous vegetables. Volunteers will be divided into two dietary intervention groups accordingly.

1. The ‘peas’ group will be asked to consume an additional 4 portions (100g each) of peas per week as part of their normal diet.

2. The ‘broccoli’ group will be asked to consume an additional 4 portions (100g each) of a special variety of broccoli, selectively grown for its enhanced glucosinolate content, per week as part of their normal diet.

Volunteers will be asked to modify their diets for a total of 12 months. This is to enable us to detect any long-term changes in gene expression and to coincide with the routine TRUS examinations every 6 months. Volunteers can choose which day to eat the vegetables, but will be asked to keep a record of when the vegetables were eaten.

Should the volunteers wish to go on holiday during the 12-month period, they will not be expected to adhere to the intervention. We will ask the volunteers to inform us of their planned holiday dates so that we can keep a record. We will ask them to avoid booking holidays for more than four weeks at a time or two weeks either side of their visits to the clinic and not within a month of the final assessment.

*Supply of Vegetables*

The broccoli will be grown and harvested by Olga Ltd, Boston, Lincolnshire, and processed by Christian Salvesen, Bourne, Lincolnshire. The broccoli is a hybrid cultivar, conventionally crossed with a wild species that has an enhanced level of the glucosinolate called glucoraphanin (precursor of sulforaphane). Samples will be packaged into 100g portions and stored frozen at -18^o^C until cooked by the volunteer. Frozen peas will be bought from a supermarket. Both types of frozen vegetable will be delivered to the volunteers on a regular basis by a member of the study team or Polymek, a temperature controlled delivery service, at a time convenient to the volunteer. Should any volunteer not own a freezer, IFR will loan a PAT tested freezer to the volunteer for the duration of the study.

*Cooking Guidelines for Frozen Vegetables*

Volunteers will be provided with a steamer and given a demonstration of how to cook the vegetables by the Diet Cooks at HNU. Portions of broccoli will be steamed for 4-5 minutes and portions of peas will be steamed for 2-3 minutes. It is important that volunteers do not over cook the broccoli as this will result in delayed absorption of isothiocyanates produced. Previous studies conducted at IFR have demonstrated cooking levels required to maximize the level of ITCs ingested (unpublished data). Levels of glucosinolate and the equivalent isothiocyanates in the enriched broccoli hybrid have been measured previously at IFR, Mithen *et al* (2003). The levels correspond to a 3-4 fold increase compared to standard supermarket broccoli.

**SAMPLE ANALYSIS**

*Blood Samples*

A 10ml sample of blood will be taken from which genomic DNA will be extracted using the QIAamp DNA Mini kit protocol (Qiagen Inc). Samples will be doubly encrypted to protect the volunteer’s identity. DNA samples extracted from whole blood will be amplified using real-time PCR using the method of Cotton *et al* (2000), to determine the presence or absence of GSTM1 and GSTT1 genotypes. The frequency of variant alleles for GSTPi will also be determined. A 5ml sample of blood will be collected at baseline and at each visit to the urology clinic. This will be tested for the level of PSA, a protein that the prostate produces. PSA level will be determined by the Biochemistry department at the NNUH.

*Urine Samples*

Total volume of urine collected during 0-24 hours post broccoli consumption will be recorded and sub samples of 20ml will be stored at –20°C until analysis. The remaining urine will be discarded. A validated liquid chromatography-tandem electrospray ionisation mass spectrometry method will be used to measure the concentrations of the following ITCs in urine samples: Iberin and sulforaphane and their glutathione, cysteine-glycine, cysteine and N-acetyl cysteine conjugates. Quantification by internal standardisation, using authentic calibration standards for each analyte, such that is standard practice in our laboratory will also be used (method in publication).

*Biopsy Samples*

*Biopsies 1-6 Hybridised onto Affymetrix Microarrays*

Two from the first sample collection day, two from the second sample collection day and two from the third sample collection day, one from each lobe.

*Biopsies 7-12 – Confirmation of Expression by RT-PCR*

Two from the first sample collection day, two from the second sample collection day and two from the third sample collection day, one from each lobe.

Biopsies will be placed in RNA later and snap frozen in liquid nitrogen. Total RNA will be extracted directly from the prostate biopsies following snap freezing in RNALater™ (Ambion). This product ‘freezes’ gene transcription and stabilises the RNA present so that it can be extracted at a later stage. Total RNA isolation will be performed using RNeasy kits (Qiagen) according the manufacturer’s instructions; these are used routinely for the extraction and purification of RNA. Preliminary investigation has demonstrated that sufficient RNA of high quality can be obtained from a single needle biopsy sample.

The RNA extracted from the biopsy samples will be concentrated and the quality assessed using Agilent technologies lab-on-a-chip RNA 6000 nano assay. RNA of sufficient quality will be hybridised onto human Affymetrix U133Plus 2.0 probe arrays at the School of Biosciences, University of Nottingham. The arrays carry >47,000 transcripts, expression profiling will be performed for each transcript and the data produced will be analysed using market-leading software. Gene transcript levels in each test sample will be normalised against the values for the standard. We are particularly interested in the expression of genes that are known to respond to treatment with ITCs *in vitro* and/or in animal models.

*TaqMan Real-Time RT-PCR Assay*

Target gene mRNA levels will be determined by real-time RT-PCR using the ABI prism 7700 Sequence Detection System (Applied Biosystems, Foster City, CA). The threshold cycle (Ct) is defined as when the fluorescence signal reaches ten-times the noise signal level (baseline). Forward and reverse primers, and the fluorogenic TaqMan probes for target genes, will be designed using Primer Express Software (Applied Biosystems). Sequence homology of selected oligomers will be checked using a National Center for Biotechnology Information BLAST search to ensure that sequences will be specific to target genes. Primer and probe sets will be obtained from Sigma-Genosys, The Woodlands, TX and Applied Biosystems, respectively.

A number of TaqMan assays have already been developed and validated at IFR, and some of these are for genes that have been shown (by IFR and others) to respond *in vitro* to treatment with SFN at the level of transcription. These genes include: Thioredoxin reductases (*TR1* and *TR*2), quinone reductase (*QR*1), glutathione-S-transferase A1 (*GSTA1*), UDP-glucuronosyltransferase (*UGT1A1*), cyclooxygenase-2 (*COX2*), multidrug resistance protein-2 (*MRP-2*), Nuclear Factor Kappa B *(NF-B)* and Kruppel-like Factor 4 *(KLF4).*

**STATISTICAL ANALYSIS**

*Statistical Power*

It is not possible to calculate the power for differences in gene expression between the two intervention groups because to date there are no relevant publications that describe the effects of ITCs on gene expression *in vivo*. This is a pilot study designed to provide data from which to calculate power for future studies in this field. The nature of a power calculation is to obtain the probability of detecting a difference that is true as opposed to a false negative result. It is arguable as to the appropriateness of such calculations when applied to Affymetrix gene expression data because the number of genes is so large (>47,000). Therefore we are more concerned with controlling false positive results (type I error).

All array data will be analysed using a simple linear model comparing significant differences in gene expression between broccoli and pea treatment groups. Impact of genotype will also be included, combining regression analysis with analysis of group differences. Pair wise comparisons between time points (baseline, mid and end points) and treatment (broccoli or peas) will be made by dividing by signal intensity to give a fold induction for each gene. Advice will be sought from Dr. Rob Foxall, Biostatistician at the Institute of Food Research, Norwich.

**ETHICAL CONSIDERATIONS**

**Food safety**: IFR has standard operating procedures for the storage and delivery of food (broccoli and peas) to study participants. Those procedures will be adhered to and are integral to IFR's recognition as ISO 9001:2000 compliant and constitute part of our process working to the standards of Good Clinical Practice and Environmental Health Guidelines.

**Toxicity:** There is no evidence from animal or human studies that broccoli is harmful. However, toxic effects of indolyl glucosinolate have been identified in animals, causing goitre in animals with a deficiency in dietary iodine, Stroewsand, (1995). The mechanism of this action is not well understood and there is no evidence of such acute effects in humans with intakes of glucosinolate below 50 mg/day. This level far exceeds the concentration consumed by volunteers in this study.

**Progression of high-grade PIN to cancer:** During the course of the study it is estimated that 30-40% of the population may develop prostate cancer. These volunteers will be withdrawn from the study with diplomacy and tact, the non-medical members of the study team being guided by the clinicians.

**REFERENCES**

Chiao JW, Chung F-L, Kancherla R, Ahmed T, Mittelman A and Conaway CC (2002). Sulforaphane and its Metabolites Mediate Growth Arrest and Apoptosis in Human Prostate Cancer Cells, *International Journal of Oncology*, **20**, 631-636

Cotton SC, Sharp L, Little J and Brockton N (2000). Glutathione-S-Transferase Polymorphisms and Colorectal Cancer: A HuGE Review, *American Journal of Epidemiology*, **151** (1) 7-31

Fenwick, GR, Heaney RK and Mullin JW, Glucosinolates and their Breakdown Products in Food and Food Plants, (1983). *CRC Critical Reviews in Food Science and Nutrition*, **18**, 123-201

Gamet-Payrastre L, Li P, Lumeau S, Cassar G, Dupont M-A, Chevolleau, Gasc N, Tulliez J and Terce F (2000). Sulforaphane, a Naturally Occurring Isothiocyanate, Induces Cell Cycle Arrest and Apoptosis in HT29 Colon Cancer Cells. *Cancer Research****,* 60**, 631-636

Giovannucci E, Rimm EB, Liu Y, Stampfer MJ and Willett WCA, (2003). A Prospective Study of Cruciferous Vegetables and Prostate Cancer. *Cancer Epidemiology, Biomarkers and Prevention,* **12**, 1403-1409.

Hecht S, (2000). Inhibition of Carcinogenesis by Isothiocyanates. *Drug Metabolism Reviews*, **32**, 395-411

Hintze K, Keck A-S, Finlay, J and Jeffery E, (2003). Induction of Hepatic Thioredoxin Reductase Activity by Sulforaphane Both in Hepa1c17 Cells and in Male Fisher 344 Rats. *Journal of Nutritional Biochemistry*, **14**, 173-179

Key TJ, Silcocks PB, Davey GK, Appleby PN and Bishop, DT, (1997). A Case-control of Diet and Prostate Cancer. *British Journal of Cancer*, **76** (5), 678-687

Kristal AR, Brassica Vegetables and Prostate Cancer Risk: A Review of the Epidemiologic Evidence, (2000). *Pharmaceutical Biology*, **40**, 55-58.

Mithen RF, Dekker M, Verkerk R, Rabot S and Johnson IT, (2000). The Nutritional Significance, Biosynthesis and Bioavailability of Glucosinolates in Human Foods. *The Journal of the Science of Food and Agriculture*, **80**, 967-984.

Mithen RF, Faulkner K, Magrath R, Rose P, Williamson G and Marquez J (2003). Development of Isothiocyanate-enriched Broccoli, and its Enhanced Ability to Induce Phase 2 Detoxification Enzymes in Mammalian Cells. *Theoretical and Applied Genetics*, **106**, 727-734.

Stroewsand G, (1995). Bioactive Organosulfur Phytochemicals in Brassica oleracea Vegetables – A Review. *Food Chemistry Toxicology,* **33,** 537-543

Surh Y-J, (2003). Cancer Chemoprevention with Dietary Phytochemicals. *Nature Reviews*, **3**, 768-780.

Verhoeven D, Verhagen H, Goldbohn RA, Van den Brandt PA, Van Poppel G, (1997). A Review of the Mechanisms Underlying Anticarcinogenicity by Brassica Vegetables. *Chemico-Biological Interactions*, **103**, 79-129
