# Supplementary material for: Broccoli Consumption Interacts with GSTM1 to Perturb Oncogenic Signalling Pathways in the Prostate
Source: PLoS One. 2008 Jul 2;3(7):e2568. doi: 10.1371/journal.pone.0002568 (PMC2430620; doi:10.1371/journal.pone.0002568)
Supplement: Figure S1 — CONSORT flow diagram. (0.03 MB DOC) [file pone.0002568.s009.doc]

**Consort Flow Diagram Broccoli, Peas and PIN Study**

Assessed for eligibility (n=32)

**Enrollment**

Excluded (n= 10)

Not meeting inclusion criteria

(n= 0)

Refused to participate

(n= 10)

Other reasons

(n= 0)

**Allocation**

**Analysis**

**Follow-Up**

Analysed (n= 7)

Excluded from paired analysis (n= 4)

Give reasons: RNA from baseline sample insufficient quality

Lost to follow-up (n= 0)

Give reasons

Discontinued intervention

(n= 1)

# Give reasons:

Progression to PC on second biopsy

Allocated to **peas** intervention

(n= 9)

Received allocated intervention

(n= 8)

Did not receive allocated intervention

(n= 1)

Give reasons: Progression to PC on first biopsy

Lost to follow-up (n=0)

Give reasons

Discontinued intervention

(n= 0)

Give reasons:

Allocated to **broccoli** intervention

(n= 13)

Received allocated intervention

(n= 13)

Did not receive allocated intervention

(n= 0)

Give reasons:

Analysed (n= 13)

Excluded from paired analysis (n= 6)

Give reasons: RNA from baseline samples insufficient quality

Group allocation matched by genotype
