# Supplementary material for: Broccoli Consumption Interacts with GSTM1 to Perturb Oncogenic Signalling Pathways in the Prostate
Source: PLoS One. 2008 Jul 2;3(7):e2568. doi: 10.1371/journal.pone.0002568 (PMC2430620; doi:10.1371/journal.pone.0002568)
Supplement: Figure S2 — Modification of TGFβ1 by SF and iodoacetamide. (0.07 MB DOC) [file pone.0002568.s010.doc]

**Figure S2.** Modification of TGFβ1 by SF and iodoacetamide to result in mass addition of b series ions of 217. Addition of SF to the N-terminus residue of TGFβ1 results in a thiourea derivative. Subsequent reaction with iodoacetamide gives a mixture of isomeric carbamimidoylsulfanylacetamides, which undergo cyclisation and loss of NH_3_ in the mass spectrometer to give the corresponding iminothiazolidinones, resulting in a mass addition to TGFβ1 of 217.
