# Supplementary material for: Broccoli Consumption Interacts with GSTM1 to Perturb Oncogenic Signalling Pathways in the Prostate
Source: PLoS One. 2008 Jul 2;3(7):e2568. doi: 10.1371/journal.pone.0002568 (PMC2430620; doi:10.1371/journal.pone.0002568)
Supplement: Text S1 — Approval from Norwich Research Ethics Committee. (0.07 MB DOC) [file pone.0002568.s008.doc]

**Text S4.** Approval from Norwich Ethics Committee.

Norwich Research Ethics Committee

c/o The Norfolk & Norwich University Hospital NHS Trust

First Floor

Aldwych House

57 Bethel Street

Norwich

NR2 1NR

02 March 2005

Professor Richard Mithen

Head Of Phytochemicals and Health

Institute Of Food Research, Norwich

Norwich Research Park,

Colney Lane

Norwich

NR4 7UA

Dear Professor Mithen

| ***Full title of study:*** | ***A Human Intervention Trial Studying Gene Expression In High-Grade Prostatic Intraepithelial Neoplasia Following Consumption Of Broccoli Or Peas*** |
| --- | --- |
| ***REC reference number:*** | ***05/Q0101/9*** |
| ***Protocol number:*** | ***5: 18/2/2005*** |
|  |  |

Thank you for your letter of 18 February 2005, responding to the Committee’s request for further information on the above research and submitting revised documentation.

The Chair together with Mrs Pam Keeley and Mr Gary Simons has considered the further information on behalf of the Committee.

## **Confirmation of ethical opinion**

On behalf of the Committee, I am pleased to confirm a favourable ethical opinion for the above research on the basis described in the application form, protocol and supporting documentation as revised.

However, the Committee would advise a modification to the text in the Letter of Invitation (Annex 1) and the Letter to the GP (Annex 4) to clarify the full and short title of the study.

# Conditions of approval

The favourable opinion is given provided that you comply with the conditions set out in the attached document. You are advised to study the conditions carefully.

The favourable opinion applies to the research sites listed on the attached form.

## **Approved documents**

The final list of documents reviewed and approved by the Committee is as follows:

| **Document Type:** | **Version:** | **Dated:** | **Date Received:** |
| --- | --- | --- | --- |
| Application | 4: Parts A, B: Section 4 & 5, C | 26/01/2005 | 26/01/2005 |
| Investigator CV | Richard Mithen |  | 26/01/2005 |
| Investigator CV | Amy Gasper |  | 26/01/2005 |
| Protocol | 5 | 18/02/2005 | 18/02/2005 |
| Covering Letter |  | 26/01/2005 | 26/01/2005 |
| Peer Review | IFR Human Research Governance Committee | 23/11/2004 | 26/01/2005 |
| Peer Review | IFR - HRGC | 08/12/2005 | 26/01/2005 |
| Peer Review | East Norfolk & Waveney RGC | 11/01/2005 | 26/01/2005 |
| Statistician Comments | Dr Rob Foxall |  | 26/01/2005 |
| Letters of Invitation to Participants | 5: with reply slip  (Annex 1) | 18/02/2005 | 18/02/2005 |
| GP/Consultant Information Sheets | 4 | 26/01/2005 | 26/01/2005 |
| Participant Information Sheet | TRUS: April 2004 | 01/04/2004 | 26/01/2005 |
| Participant Information Sheet | 3: Collection, storage & use of tissue | 01/02/2003 | 26/01/2005 |
| Participant Information Sheet | 5: Annex 2 | 18/02/2005 | 18/02/2005 |
| Participant Consent Form | 3: Collection & storage of human tissue | 01/02/2003 | 26/01/2005 |
| Participant Consent Form | 4 | 26/01/2005 | 26/01/2005 |
| Response to Request for Further Information |  | 18/02/2005 | 18/02/2005 |
| Patient withdrawal letter | 4 | 26/01/2004 | 26/01/2005 |
| Indemnity |  | 26/01/2004 | 26/01/2005 |
| Volunteer Health Declaration & Medical Declaration Form | 5 | 01/03/2004 | 26/01/2005 |
| 7-day estimated food intake diary | 4 | 26/01/2005 | 26/01/2005 |
| Vegetable record sheet | 4 | 26/01/2005 | 26/01/2005 |

## **Management approval**

The study should not commence at any NHS site until the local Principal Investigator has obtained final management approval from the R&D Department for the relevant NHS care organisation.

### Membership of the Committee

The members of the Ethics Committee who were present at the meeting are listed on the attached sheet.

## **Notification of other bodies**

The Committee Administrator will notify the research sponsor and the R&D Department for NHS care organisation that the study has a favourable ethical opinion.

### Statement of compliance

*The Committee is constituted in accordance with the Governance Arrangements for Research Ethics Committees (July 2001) and complies fully with the Standard Operating Procedures for Research Ethics Committees in the UK.*

# 05/Q0101/9 Please quote this number on all correspondence

With the Committee’s best wishes for the success of this project,

Yours sincerely,

**Dr Mike Sampson**

##### Chair

E-mail: katheriner.norton@nnuh.nhs.uk

#### Enclosures List of names and professions of members who were present at the

#### meeting and those who submitted written comments

#### Standard approval conditions

Site approval form (SF1)

Cc	Amy Gasper

David Hughes, Chair IFR HRGC	Ref: IFR07/2004

East Norfolk & Waveney Research Governance Committee	Ref: 2004IFR04
